# Integrative analysis of the steroidal alkaloids distribution and biosynthesis of bulbs *Fritillariae Cirrhosae* through metabolome and transcriptome analyses

**DOI:** 10.1186/s12864-022-08724-0

**Published:** 2022-07-14

**Authors:** Qiuxia Lu, Rui Li, Jiaqing Liao, Yuqin Hu, Yundong Gao, Mingcheng Wang, Jian Li, Qi Zhao

**Affiliations:** 1 College of Food and Biological Engineering, Chengdu University, Chengdu, 610106 China; 2 Institute of Cancer Biology and Drug Discovery, Chengdu University, Chengdu, 610106 China; 3 Engineering Research Center of Sichuan-Tibet Traditional Medicinal Plant, Chengdu, 610106 China; 4 College of Pharmacy, Chengdu University, Chengdu, 610106 China; 5 Aba County Shenhe Agricultural Development Co. LTD, Aba County, 624600 China; 6 CAS Key Laboratory of Mountain Ecological Restoration and Bioresource Utilization & Ecological Restoration and Biodiversity Conservation Key Laboratory of Sichuan Province, Chengdu Institute of Biology, Chinese Academy of Sciences, Chengdu, 610041 China; 7 Institute for Advanced Study, Chengdu University, No. 2025 Chengluo Road, Chengdu, 610106 China; 8 State Key Laboratory of Southwestern Chinese Medicine Resources, Chengdu, 610106 China; 9 School of Basic Medical Sciences, Chengdu University, Chengdu, 610106 China

**Keywords:** *Fritillaria cirrhosa*, Liliaceae, Steroidal alkaloid biosynthesis, Metabolomics, Transcriptome

## Abstract

**Background:**

Bulbus *Fritillariae Cirrhosae* (BFC) is an endangered high-altitude medicine and food homology plant with anti-tumor, anti-asthmatic, and antitussive activities as it contains a variety of active ingredients, especially steroidal alkaloids. Bulbus *Fritillariae Thunbergia* (BFT) is another species of *Fritillaria* that grows at lower altitude areas. Production of plant-derived active ingredients through a synthetic biology strategy is one of the current hot topics in biological research, which requires a complete understanding of the related molecular pathways. Our knowledge of the steroidal alkaloid biosynthesis in *Fritillaria* species is still very limited.

**Results:**

To promote our understanding of these pathways, we performed non-target metabolomics and transcriptome analysis of BFC and BFT. Metabolomics analysis identified 1288 metabolites in BFC and BFT in total. Steroidal alkaloids, including the proposed active ingredients of *Fritillaria* species peimine, peimisine, peiminine, etc., were the most abundant alkaloids detected. Our metabolomics data also showed that the contents of the majority of the steroidal alkaloids in BFC were higher than in BFT. Further, our comparative transcriptome analyses between BFC and BFT identified differentially expressed gene sets among these species, which are potentially involved in the alkaloids biosynthesis of BFC.

**Conclusion:**

These findings promote our understanding of the mechanism of steroidal alkaloids biosynthesis in Fritillaria species.

**Supplementary Information:**

The online version contains supplementary material available at 10.1186/s12864-022-08724-0.

## Background

*Fritillaria* (Liliaceae) is a perennial herb genus with more than 130 species found in North America, the Mediterranean, and Asia [1]. Many species of *Fritillaria* have been used as herbal remedies in many countries' folk medicines for a long history [2, 3]. Steroidal alkaloids are specialized metabolites found mainly in the families of *Liliaceae* [4]. In China, the dried bulbs derived from various *Fritillaria* species (called "Beimu" in Chinese) are exploited as medicine and food homology plants because of their anti-tumor, anti-asthmatic, and antitussive activities [5–7]. Among the different species of *Fritillaria*, Bulbus *Fritillariae cirrhosae* (BFC) and *Fritillariae Thunbergia* (BFT), which are called “Chuan-Bei-Mu” and “Zhe-Bei-Mu” in Chinese, and distributed at different altitudes, are considered as two most valuable species for their superior therapeutic effects on cough and asthma, compared with other *Fritillaria* species [8] (More than 100 kinds of Chinese patented medicines use BFC as the raw material [9]. BFC can be derived from six original plants, *Fritillaria cirrhosa* D.Don (Liliaceae), *Fritillaria wabuensis* (S.Y.Tang et S.C.Yue) Z.D.Liu, S.Wang et S. C. chen (Liliaceae), *Fritillaria unibracteata* Hsiao et K.C.Hsia (Liliaceae), *Fritillaria przewalskii* Maxim (Liliaceae), *Fritillaria delavayi* Franch (Liliaceae), and *Fritillaria taipaiensis* P.Y.Li. (Liliaceae) [5, 10]. Wild BFC resources are endangered due to long-term excessive mining [11, 12]. The estimated annual market demand for BFC is about 2,000 tons, with a gap of 1,900 tons [3]. BFT is a member of the geo-authentic crude drugs in Zhejiang Province, its production ranks first in the commodity Bulbus *Fritillaria* and has been approved as a national health functional food for relieving cough and reducing sputum [13]. Steroidal alkaloids are reported to be the main parts of BFC that give it its health benefitsBFC [5, 14]. Dissecting the pathways responsible for the BFC alkaloids synthesis is conducive to producing these active ingredients in biotechnological manner, especially synthetic biology. Until now, many efforts have been made to identify each of the enzymes involved in synthesizing the steroidal alkaloids [15]. However, our understanding of the whole picture of alkaloid synthesis pathways in BFC is still limited. Recently, a few studies have been carried out to investigate the molecular mechanisms that control the steroidal alkaloid biosynthesis in *Fritillaria* species. For example, Zhao et al. performed transcriptome analysis to map the gene expression profile of in vitro regenerated BFC [16]. Kumar et al. identified the essential genes and regulatory transcription factors (TFs) of imperialine biosynthesis in *Fritillaria roylei* Hook using comparative de novo transcriptome analysis [17]. The molecular basis of organ-specific expression of isosteroidal alkaloids biosynthesis in *F. roylei* Hook was investigated by transcriptome analysis [18]. In the current study, we conducted the first metabolomics and transcriptomics combined analysis to systematically investigate the characteristics of steroidal alkaloids synthesis in the five species of BFC and BFT, another *Fritillaria* species distributed in the lower altitude areas. Our findings from the current study promoted our understanding of the mechanism of steroidal alkaloids biosynthesis in *Fritillaria* species.

## Results

### Metabolome data quality analysis

**Fig. 1:**
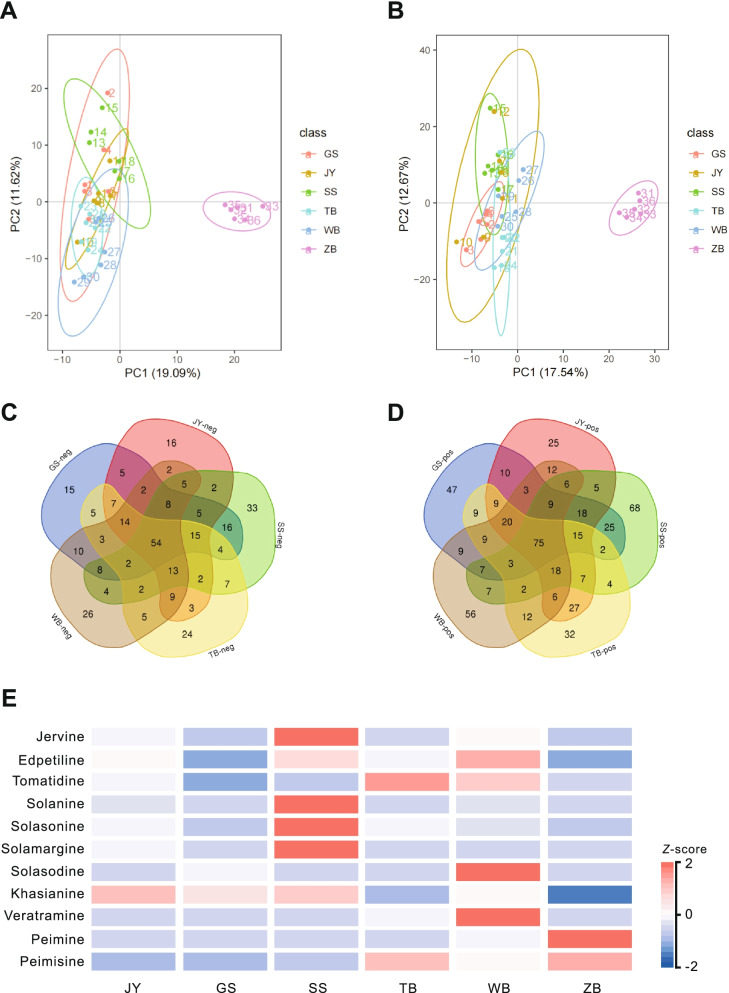
Principal component analysis of the metabolite quantification and heat map analysis of 11 differential steroidal alkaloid metabolites in BFC andBFT*.* **A** PCA derived from the LC–MS/MS (ESI-); **B** PCA derived from the LC–MS/MS (ESI +); **C-D** Venn diagram depicting the shared and unique differentially accumulated metabolites (DAMs) between pairs of BFC and BFT*;* **E** The values of differential metabolites were normalized and shown as a color scale. The high and low metabolite levels were represented as reddish and blueish scales. (*Fritillaria* przewalskii (GS), *Fritillaria cirrhosa* D. Don (JY), *Fritillaria delavayi* (SS), *Fritillaria taipaiensis* (TB), *Fritillaria unibracteata* (WB) and *Fritillaria thunbergii* (ZB))

As shown in Fig. 1A-B, while the biological replicates of each species clustered in different areas in the PCA analysis, there was an evident separation between BFC and BFT, indicating significant differences in terms of their metabolites. A higher PC1 value showed more genetic variation among varieties. PC1 and PC2, two principal components, were 16.77% and 12.42% in the positive ion mode and 17.69% and 11.23% in the negative ion mode, respectively.

### Comparison of the metabolites between BFC and BFT

A total of 1288 metabolites were detected from BFC and BFT by conducting liquid chromatography-tandem mass spectrometry (LC − MS/MS) (File S1 and File S2). Alkaloids, saponins, phenylpropanoids, carboxylic acids and their derivatives, flavonoids, and organic acids and their derivatives were the most common metabolites.

Differentially accumulated metabolites (DAMs) were screened based on fold-change (FC) ≥ 2 or ≤ 0.5 among the metabolites with a VIP value > 1 and a *P*-value < 0.05. The results were shown in File S3 and the Venn diagrams (Fig. 1C-D). A total of 443 DAMs, including 306 upregulated and 137 downregulated, were identified between *F. przewalskii* (GS) and *F. thunbergii* (ZB). Similarly, there were 427 DAMs (108 upregulated and 207 downregulated) between *F. cirrhosa* D. Don (JY) and *F.thunbergii* (ZB). 451 DAMs (288 upregulated and 163 downregulated) were identified between *F. delavayi* (SS) and *F. thunbergii* (ZB). 419 DAMs (230 upregulated and 189 downregulated) were found between *F. taipaiensis* (TB) and *F. thunbergii* (ZB). We also identified 421 DAMs, including 247 downregulated and 174 upregulated, between *F. unibracteata* (WB) and *F. thunbergii* (ZB). KEGG enrichment analysis showed that these differential metabolites between BFC and BFT were mainly enriched in “Metabolic pathways,” “Biosynthesis of specialized metabolites,” “Biosynthesis of amino acids,” “Aminoacyl-tRNA biosynthesis,” and “Pyrimidine metabolism,” etc. (Fig. S1).

### Identification of differentially produced alkaloids in BFC compared to BFT

To find the significant differential metabolites, the top 20 differentially (up and down) produced metabolites in BFC and BFT were analyzed. The results indicated that the primary differential metabolites in six species were steroidal alkaloids, tropine alkaloids, pyridines alkaloids, indole alkaloids, isoquinoline alkaloids, organic amines alkaloids, etc. (Table S1). Steroidal alkaloids contained peimine, peimisine, peiminine, jervine, tomatidine, solanine, solasonine, solamargine, solasodine, and veratramine, as well as two undescribed compounds, i.e.edpetiline, and khasianine. As shown in Fig. 1E, the content of solamargine, jervine, solanine, solasonine, solasodine, edpetiline, khasianine, veratramine, and tomatidine in BFC is significantly higher than that in BFT. Moreover, the highest contents of these compounds were observed in *F. delavayi* (SS). Nevertheless, peimine and peimisine were found to be higher in BFT. There was little difference in the content of peiminine between BFC and BFT (Table S2).

### De novo assembly and functional annotation of BFC and BFT unigenes

Eighteen RNA-Seq libraries were constructed and sequenced for the six species, designated JY_S1-S3, GS_S1-S3, SS_S1-S3, TB_S1-S3, WB_S1-S3, and ZB_S1-S3, respectively. Overall, each library had an average of 23.43 million raw reads, ranging from 20.64 million to 26.25 million (Table S3). Adapters, empty reads, and low-quality sequences were removed from the raw reads before being analyzed for their genetic content. Consequently, each library generated an average of 23.02 million clean reads, totaling 6.91 Gb in size and approximately 49.71% GC content. This study used NCBI-NR, NCBI-NT, PFAM, KOG/COG, GO, and KEGG to annotate gene function (Fig. S2A). A total of 31,342 genes were annotated via GO clustering analysis. These genes were clustered into three major categories and 43 subcategories, including 5 cellular components, 12 molecular functions, and 26 biological processes. In the biological process, "metabolic process," "biological regulation," and "cellular process" are the main functional categories (Fig. S2B and File S4).

Twenty-six thousand nine hundred seventy genes were annotated for 295 KEGG metabolic pathways in the five *F. cirrhosa* species and *F.thunbergii*. Among them, "Steroid biosynthesis" (55 unigenes), "Ubiquinone and other terpenoid-quinone biosynthesis" (102 unigenes), "Terpenoid backbone biosynthesis" (131 unigenes), "Diterpenoid biosynthesis" (91 unigenes), and "Sesquiterpenoid and triterpenoid biosynthesis" (13 unigenes) are related to the regulation of alkaloids biosynthesis (Fig. S2C and File S4).

### Comparative analysis of DEGs between BFC and BFT

DEGs were further identified by comparing five species of BFC and BFT. Specifically, 11,577 DEGs, including 6304 upregulated and 5273 downregulated genes, were identified between *F. przewalskii* (GS) and *F. thunbergii* (ZB). Similarly, there were 10,847 DEGs (5139 upregulated and 5708 downregulated) between *F. cirrhosa* D. Don (JY) and *F. thunbergii* (ZB), 16,504 DEGs (9054 upregulated and 7446 downregulated) between *F. delavayi* (SS) and *F. thunbergii* (ZB), 19,951 DEGs (10,536 upregulated and 9388 downregulated) between *F. taipaiensis* (TB) and *F. thunbergii* (ZB), and 18,018 DEGs (8214 downregulated and 9804 upregulated) between *F. unibracteata* (WB) and *F. thunbergii* (TB) (Fig. S3). The DEGs of BFC and BFT bulbs might indicate differences in specialized metabolites accumulation in BFC and BFT.

### Comparative functional annotation of identified DEGs in BFC and BFT

**Fig. 2:**
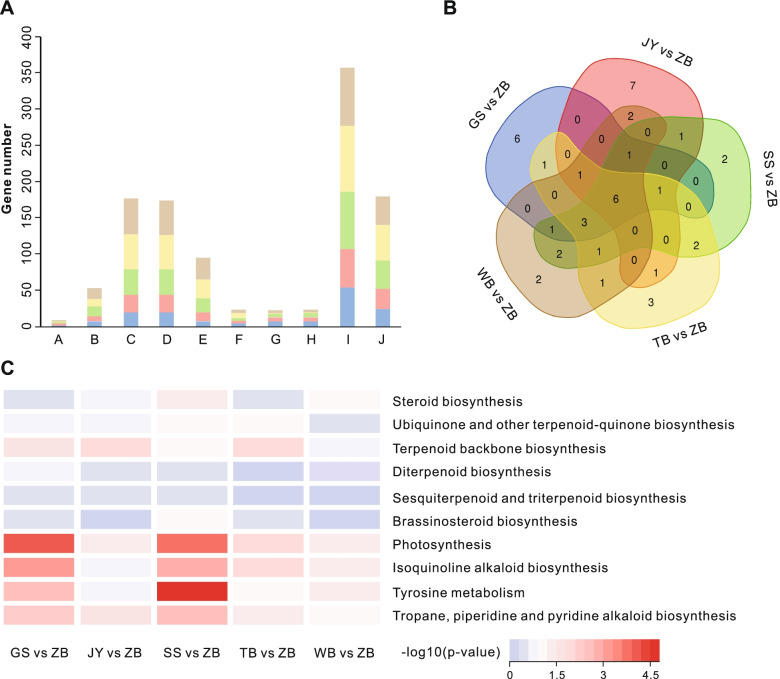
Comparative analysis of significantly enriched GO and KEGG pathways. **A** Go Term (BP) (A alkaloid biosynthetic process; B specialized metabolite biosynthetic process; C. Steroid metabolic process; D. steroid biosynthetic process; E. C21-steroid hormone metabolic process; F. sterol biosynthetic process; G. seed development; H. reproductive system development; I. photosynthesis; J. isoprenoid metabolic process; GS-blue; JY-pink; SS-green; TB-yellow; WB-gray); **B** KEGG enrichment analysis [21, 22]; **C** 10 enriched KEGG pathways enriched in BFC compared with BFT [23]

To further understand the biological activities of these genes involved in alkaloid biosynthesis, functional classifications of DEGs found among bulbs in BFC, and BFT were carried out. First, GO clustering analyses were adopted to annotate the DEGs function between the bulbs of BFC and BFT. We found 3597 GO terms enriched in at least one DEG group, and 3208 shared GO terms were enriched in all four DEG groups (File S5). We compared the enriched biological processes of these five DEG groups since we were primarily interested in producing and accumulating alkaloids. As shown in Fig. 2A, alkaloid biosynthetic process, specialized metabolite biosynthetic process, C21-steroid hormone metabolic process, sterol biosynthetic process, seed development, and reproductive system development were enriched in BFC and BFT. In addition, steroid metabolic process, steroid biosynthetic process, photosynthesis, and isoprenoid metabolic process are also the significantly enriched biological processes with the most significant number of DEGs.

Next, KEGG enrichment analysis showed 6 shared pathways enriched in all five DEG groups in the top 20 KEGG pathways (Fig. 2B, File S6). In addition, 10 enriched KEGG pathways (Fig. 2C, and Fig. S4), such as ubiquinone and other terpenoid-quinone biosynthesis, terpenoid backbone biosynthesis, isoquinoline alkaloid biosynthesis, tyrosine metabolism, and tropane, piperidine, and pyridine alkaloid biosynthesis significantly enriched in BFC. In addition, steroid biosynthesis, diterpenoid biosynthesis, and brassinosteroid biosynthesis were also enriched in BFC. These pathways are involved in the biosynthesis of the precursor substances of alkaloids. Furthermore, photosynthesis is also mainly enriched in BFC. One of the most significant environmental elements in plant existence is photosynthesis. It is not only a source of energy for plant growth and development, but it also serves as a signal to control plant growth and development. In most plants, photosynthesis has an impact on specialized metabolite synthesis. The production of alkaloids is influenced by photosynthesis [19, 20].

### Analysis and identification of candidate genes possibly involved in the steroidal alkaloids biosynthesis of BFC

**Fig. 3:**
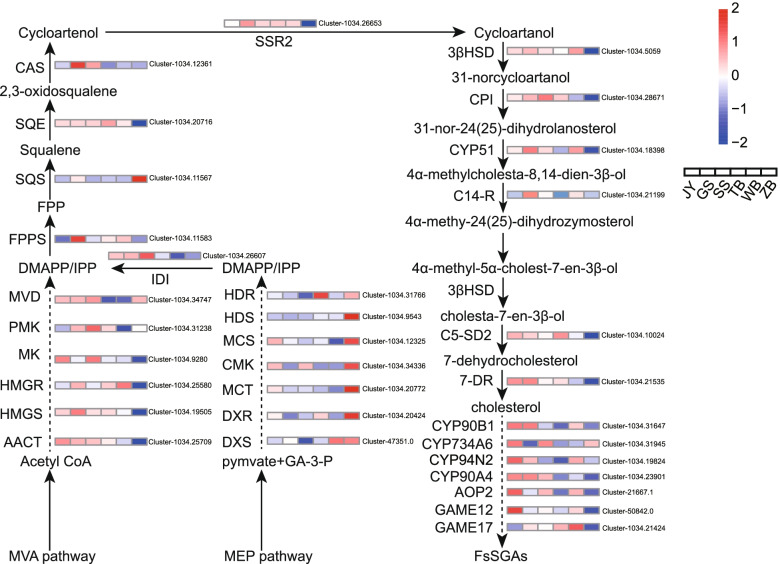
Schematic diagram of fold change in expression level of predicted structural genes in steroidal alkaloids in *Fritillaria* bulbs between six species

To identify the candidate genes that are possibly involved in the steroidal alkaloids biosynthesis pathway in *Fritillaria* species, we built a pathway diagram that included the expression heat maps for the structural gene in the six *Fritillaria* species that are predicted to be involved in the steroidal alkaloids biosynthesis pathways (Fig. 3). Methyl erythritol phosphate (MEP) and mevalonic acid (MVA) signaling pathways are essential routes involved in the biosynthesis of dimethylallyl diphosphate (DMAPP) and isopentenyl diphosphate (IPP), which are precursors of steroidal alkaloids [24]. We found that Cluster-1034.25709 (predicted to be acetyl-CoA C-acetyltransferase, AACT), Cluster-1034.19505 (predicted to be 3-hydroxy-3-methylglutaryl-CoA synthase, HMGS), Cluster-1034.25580 (predicted to be 3-hydroxy-3-methylglutaryl-CoA reductase, HMGR), Cluster-1034.9280 (predicted to be mevalonate kinase, MK), Cluster-1034.31238 (predicted to be 5-phosphomevalonate kinase, PMK), and Cluster-1034.34747(predicted to be mevalonate 5-diphosphate decarboxylase, MVD), which have been reported to participate in the DMAPP/IPP synthesis through the MVA pathway in other plants [25], were expressed higher in BFC than that in BFT (Fig. 3 and Table S4). On the other hand, the genes reported to be involved in the MEP pathway in other plants [20], including Cluster-47351.0 (predicted to be 1-deoxy-D-xylulose-5- phosphate synthase, DXS), Cluster-47351.0 (predicted to be 1-deoxy-D-xylulose-5- phosphatereducto-isomerase, DXR), Cluster-1034.20772 (predicted to be 2-C-methyl-D- erythritol-4-phosphate cytidylyltransferase, MCT), Cluster-1034.34336 (predicted to be 4-(cytidine 5-diphospho) -2-C-methyl- Derythritolkinase, CMK), Cluster-1034.12325(predicted to be 2-C-methyl- erythritol 2,4-cyclodiphosphatesynthase, MCS), Cluster-1034.9543 (predicted to be 1-hydroxy-2- methyl-2-(E)- butenyl-4-diphosphate synthase, HDS), and Cluster-1034.31766 (predicted to be 1-hydroxy-2-methyl-2-(E)- butenyl-4- diphosphate reductase, HDR) were found to be expressed higher in BFT than that of in BFC. These findings suggested that the MVA pathway and MEP pathway are likely to be responsible for the biosynthesis of the key intermediate of steroidal alkaloids in BFC and BFT, respectively.

DMAPP/IPP serves as the substrates that undergo a cascade of chemical conversions along with the formation of metabolite intermediates, such as geranyl pyrophosphate (GPP), farnesyl pyrophosphate (FPP), squalene, 2,3-oxides squalene, and cycloartenol, through terpenoid backbone biosynthesis [26]. Farnesyl pyrophosphate synthase (FPPS), squalene synthase (SQS), squalene monooxygenase (SQE), cycloartenol synthase (CAS), and sterol side chain reductase (SSR2) participated in this process. By screening the DEGs among the six species, we found that the expression level of the candidate genes for these enzymes (Cluster-1034.11583, predicted to be FPPS; Cluster-1034.11567, predicted to be SQS; Cluster-1034.20716, predicted to be SQE; Cluster-1034.12361, predicted to be CAS; Cluster-1034.26653, predicted to be SSR2) was higher in BFC, compared with BFT.

Cycloartenol serves as an important substrate for cholesterol formation, which is the precursor of steroidal alkaloids. Our analysis found that the candidate genes related to the conversion of cycloartenol to cholesterol, including Cluster-1034.5059 (predicted to be 3-beta-hydroxysteroid-dehydrogenase/decarboxylase, 3βHSD), Cluster-1034.28671 (predicted to be Cyclopropyl isomerase, CPI), Cluster-1034.18398 (predicted to be sterol 14-demethylase, CYP51), Cluster-1034.10024 (predicted to be sterol C-5 desaturase, C5-SD), and Cluster-1034.21535 (predicted to be 7-dehydrocholesterol reductase, 7-DR) gene (Table S4), were expressed higher in the BFC than in BFT.

Additional reactions, including hydroxylation, oxidation, transamination, and glycosylation, are involved in the downstream steps from cholesterol to different types of steroidal alkaloids. The candidate genes, i.e. (Cluster-1034.31647, predicted to be steroid 22-alpha-hydroxylase, CYP90B1), Cluster-1034.23901, predicted to be Steroid 23-alpha-hydroxylase, CYP90A4), (Cluster-1034.31945, predicted to be steroid 26-alpha-hydroxylase, CYP734A6), (Cluster-1034.19824, predicted to be steroid 22,26-alpha-hydroxylase, CYP94N2), (Cluster-21667.1, predicted to be 2-oxoglutarate-dependent dioxygenase, AOP2), (Cluster-1034.21424, predicted to be UDP-glucose glucosyltransferase, GAME17), and (Cluster-50842.0, predicted to be Gamma-aminobutyrate aminotransferase, GAME12) were identified in the six species. The expression level of these genes was higher in BFC than in BFT.

### Identification of transcription factors (TFs) possibly involved in the biosynthesis of steroidal alkaloids by BFC

**Fig. 4:**
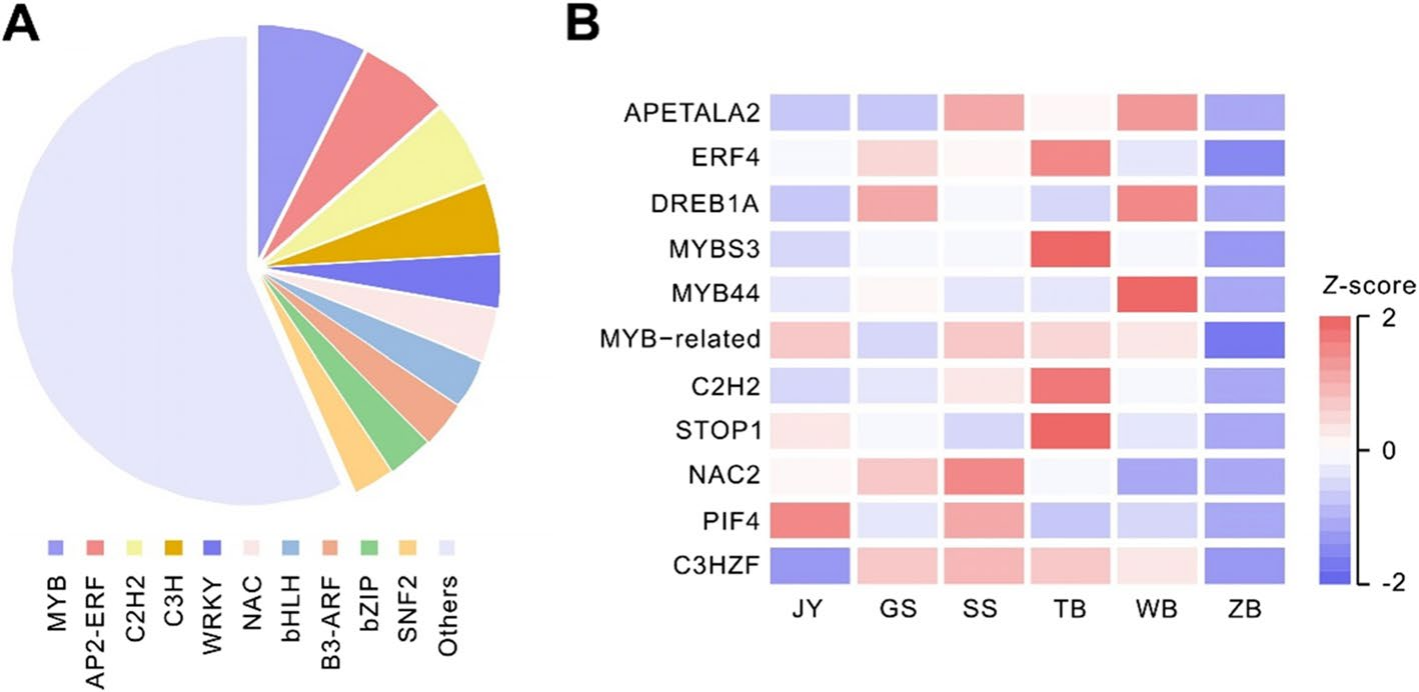
Differential expression analysis of predicted transcription factors in BFC and BFT*.* **A** Type and number of major differential transcription factors*;* **B** Heat map analysis of 10 differential regulatory genes between five varieties of BFC and BFT. The values of differential genes were normalized and shown as a color scale. The high and low transcription factor expression levels were represented as reddish and blueish scales. (*Fritillaria przewalskii* (GS), *Fritillaria cirrhosa* (JY), *Fritillaria delavayi* (SS), *Fritillaria taipaiensis* (TB), *Fritillaria unibracteata* (WB) and *Fritillaria thunbergii* (ZB))

We further screened for the transcription factors that might be associated with the synthesis of steroid alkaloids in *Fritillaria*. In total, 1957 transcripts from RNA-seq were identified with transcription factor domains, which were further categorized into 75 transcription factor families, including MYB families (151), AP2/ERF (125), C2H2 (117), C3H (97), WRKY (72) and NAC families (72), etc. (Fig. 4A). A comparative analysis of the five paired transcriptome profiles, JY vs. ZB, GS vs. ZB, SS vs. ZB, TB vs. ZB, WB vs. ZB, was conducted to identify the specific transcriptional factors possibly involved in the synthesis of steroidal alkaloids in *Fritillaria*. We found that 9 families of transcription factors, including AP2, MYB, NF, C2H2, GARP, NAC, SET, BHLH, and C3H, were expressed higher in BFC than in BFT. Among these 9 families, AP2, MYB, C2H2, NAC, BHLH, and C3H participated in regulating the synthesis of steroidal alkaloids reported in other plants. As shown in Fig. 4B, 11 candidate regulatory genes, including APETALA2 (Cluster-1034.7349), ERF4 (Cluster-1034.15804), DREB1A (Cluster-1034.19105), MYBS3 (Cluster-49397.0), MYB44 (Cluster-1034.20903), MYB-related (Cluster-1034.20351), C2H2 (Cluster-1034.36166), STOP1 (Cluster-1034.9437), NAC2 (Cluster-1034.25153), PIF4 (Cluster-1034.38121), C3HZF (Cluster-1034.17783), were significantly enriched in the five species of BFC than in BFT*.*

### qRT-PCR analysis of gene expression

To validate the transcriptome data, we performed qRT-PCR to analyze the expression of 9 structural genes and 3 regulatory genes involved in the steroidal alkaloid synthesis pathway in BFC. The results indicated that the expression patterns of 12 genes were consistent with their transcriptome expression profiles (Fig. S5).

## Discussion

Because of its antitussive, anti-inflammatory, and anti-tumor properties, BFC has long been used as a medicine and food homology plant [27, 28]. Its health benefits have been attributed to the active ingredients, especially steroidal alkaloids [10, 29]. The resources of wild BFC are becoming scarce. Bioengineering might be a feasible way to solve this problem [30–32]. However, we must first clarify the biosynthetic pathways and genes involved in BFC steroidal alkaloids biosynthesis.

Our results from the metabolome analysis showed that alkaloid and saponin were the two primary metabolites in BFC and BFT. Alkaloids contain steroidal alkaloids, tropine alkaloids, pyridine alkaloids, indoles alkaloids, isoquinoline alkaloids, organic amine alkaloids, etc. Apart from the previously reported steroidal alkaloids, such as peimine, peimisine, peiminine [10, 13, 28], we identified two steroidal alkaloids, edpetiline, and khasianine with potential therapeutic effects. Zhang et al. reported that edpetiline could alleviate lipopolysaccharide-induced inflammation and oxidative stress in RAW264.7 macrophages [33].

Other alkaloids, such as indoles, isoquinoline, tropine, and pyridine alkaloids, have been shown to have anti-inflammatory properties [34–38]. In this study, we also found indoles alkaloids (Tryptamine, Tryptophan, Tabersonine, and Vindoline), isoquinoline alkaloids (3,4-Dihydroxybenzaldehyde, Berbamine, Berberrubine, Lupinine, Palmatine, Emetine, and Tetrandrine), as well as tropine alkaloids and pyridines alkaloids (pipecolic acid, Nicotinic acid, Stachydrine, Ecgonine, Oxysophocarpine, Securinine, Pilocarpine, lupinine, and Tropine), in *Fritillaria* species analyzed. The content of these alkaloids is higher in BFC than in BFT. These compounds contribute to the anti-inflammatory activities of BFC.

Saponins are less studied metabolites of BFC. A recent report suggested that saponins contribute to the pharmacological activities of BFC [13]. This study found that BFC and BFT contained abundant steroid saponins and triterpenoid saponins, including timosaponin BII, protoClusterscin, astragaloside IV, chikusetsusponin Iva, polyphyllin II, polyphyllin VI, timosaponin A-III, timosaponin A1, trillin, and liriopemuscaribaily saponins. Moreover, their contents in BFC were significantly higher in *F. thunbergii*. The discovery of new metabolites is helpful to elucidate further the material basis of the effect of BFC and BFT. Our data provided new insights into understanding the active substances of BFC.

Further differential metabolite analysis showed that steroidal alkaloids were the major differentially produced alkaloids between and BFC and BFT. We found that imperialine, peimine, and edpetiline were rich in BFC [39, 40]*.* In contrast, peimisine and peiminine were higher in *F. thunbergii* than in BFC, consistent with the previously reported data [41]. According to our data, most of the steroidal alkaloid content in BFC was higher than in *F. thunbergii*, which might be why BFC is more effective than BFT. In plants, the terpenoid skeletons are synthesized by DMAPP/IPP via the MVA/MEP pathway [42]. In this study, six genes predicted to be involved in DMAPP/IPP synthesis via the MVA pathway were found to be highly expressed in BFC, while another set of genes predicted to be involved in the MEP pathway was expressed higher in *F. thunbergii*. Thus, we inferred that the MVA pathway is the primary route responsible for DMAPP/IPP synthesis in BFC, which is consistent with the results from another recent study [17]. On the other hand, the enzymes in the MEP pathway are more likely to control how DMAPP/IPP is synthesized in BFT. We don’t know much about the genes involved in the steps after cholesterol that lead to the production of steroidal alkaloids in BFC. Steroidal alkaloids can be biosynthesized from cholesterol via a series of hydroxylation or oxidation reactions of cholesterol at C-22 and C-26, hydroxylation at C-23, and transamination at C-26 [43]. CYP90B1 from *Arabidopsis thaliana* has been reported to catalyze steroid hydroxylation at the C-22 position [44]. CYP90A4 can catalyze hydroxylation at C-23 in *Oryza sativa* L. [45]. CYP734A6 was reported to mediate a C-26 hydroxylation reaction in *Solanum lycopersicum* and *A. thaliana* [46]. The steroid C-26 hydroxylase/oxidase (CYP94N1) activity was previously confirmed in *Veratrum californicum *[15]. AOP2, GAME12, and GAME 17 have been shown to introduce the amine group at C-26 of cholesterol, participate in the biosynthesis of steroidal glycoalkaloids (SGA) [44]. In the present study, we identified three candidate genes (Cluster-1034.31647, Cluster-1034.23901, Cluster-1034.31945) with C-22, C23, and C-24 hydroxylase activity. The candidate with C-26 hydroxylase/oxidase activity pointed to one contig (Cluster-1034.19824). These genes were significantly upregulated in BFC. Thus, we hypothesized that the higher expression of CYP90B1, CYP734A6, CYP94N2, and CYP90A4 contributes to the accumulation of steroid alkaloids in BFC.

AP2/ERF superfamily has been shown to regulate the transcription of genes involved in alkaloid synthesis by recognizing various GC-rich boxes in their promoters in tobacco and *Catharanthus roseus* [4]. In this study, we identified a couple of candidate transcription factors, belonging to AP2/ERF, MYB, C2H2, and bHLH families, that were highly expressed in BFC. These transcription factors have been shown to directly or indirectly regulate the biosynthesis of alkaloids in *Fritillaria roylei* Hook [17, 18, 47, 48].

## Conclusion

In summary, we systematically analyzed the primary specialized metabolites and the metabolic pathways of steroidal alkaloids biosynthesis in BFC by comparing them with BFT. Our metabolomics data also showed that the contents of the majority of the steroidal alkaloids in BFC were higher than in BFT. Our comparative transcriptome analyses between BFC and BFT identified differentially expressed gene sets among these species. We identified a series of genes that might be associated with alkaloids biosynthesis regulation in *Fritillaria* species. The results from this study would be valuable to promote our understanding of the steroidal alkaloids biosynthesis pathways.

## Materials and methods

### Plant materials

The bulbs of the five species of BFC were collected in the alpine regions of northwestern China: *Fritillaria cirrhosa* D. Don (Liliaceae) in the city of Kangding (located at 30°3′44.9″N, 101°58′3.81″E, altitude 4300 m); *Fritillaria przewalskii* Maxim (Liliaceae) and *Fritillaria unibracteata* Hsiao et K.C.Hsia (Liliaceae) in Aba Tibetan Autonomous Prefecture (located at 31°17′5.53″N, 102°35′28.99″E, altitude 4200 m); *Fritillaria delavayi* Franch (Liliaceae) in Ganzi Tibetan Autonomous Prefecture (located at 29°35′38.40″N, 101°52′48.30″E, altitude 4400 m); *Fritillaria taipaiensis* P.Y.Li. (Liliaceae) in Chongqing, southwestern China (located at 31°46′26.61″N, 109°5′58.92″E, altitude 2400 m). *Fritillaria thunbergii* Miq. (Liliaceae) was collected in the city of Jinhua (located at 29°9′56.56″N, 120°43′32.27″E, altitude 770 m). The voucher specimens of *F. cirrhosa* (No. F-200725–1), *F. przewalskii* (No. F-200725–2), *F. unibracteata* (No. F-200725–3), *F. delavayi* (No. F-200725–4), *F. taipaiensis* (No. F-200725–5), and *F. thunbergii* (No. F-200725–6) were authenticated by Dr. Qi Zhao (College of Food and Biological Engineering, Chengdu University) and deposited in the Engineering Research Center of Sichuan-Tibet Traditional Medicinal Plant, Chengdu, China. We declare that the research programme complies with relevant institutional, national and international guidelines and legislation, and we have permission to collect BFC and BFT.

**Fig.5:**
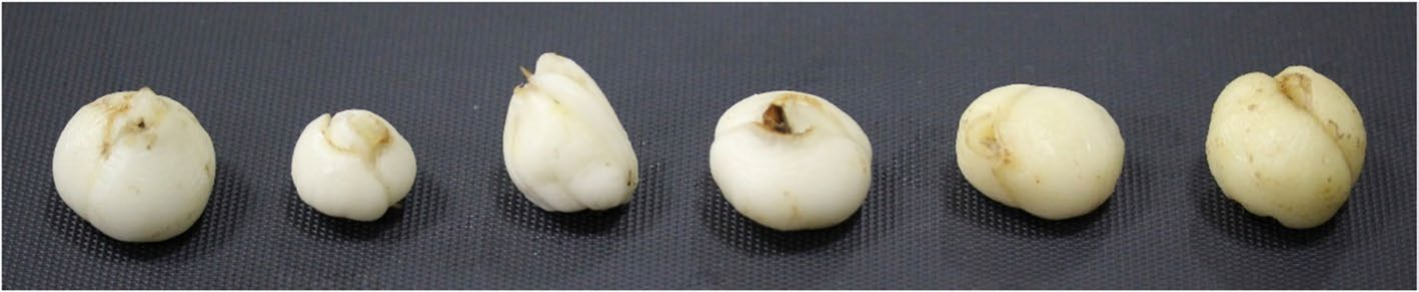
Morphological observation of the bulbs of *Fritillaria cirrhosa* and *Fritillaria thunbergii*. (From left to right are: *Fritillaria cirrhosa* D.Don (JY), *Fritillaria przewalskii* (GS), *Fritillaria delavayi* (SS), *Fritillaria taipaiensis* (TB), *Fritillaria unibracteata* (WB) and *Fritillaria thunbergii* (ZB))

The morphology of bulbs from six varieties is shown in Fig. 5. For each species, the bulbs of six individuals were randomly collected for metabolic profiling. Three biological replicates of bulb samples from each species were frozen in liquid nitrogen and stored at − 80℃ for transcriptome sequencing and reverse transcription-quantitative polymerase chain reaction (RT-qPCR) analyses. Metabolic and transcriptional profiling analyses were performed by Novogene Co., LTD. (Beijing, China).

### Metabolite extraction

Bulb homogenate was reconstituted from 100 mg of each sample with prechilled 80% methanol and 0.1% formic acid by vortexing after being ground in liquid nitrogen. Incubation of the samples on ice for five minutes followed by centrifugation for 20 min at 15,000 g (4℃). 53% methanol was used to dilute the supernatant, and the supernatant was centrifuged once more for 20 min at 15000 g **(**4 °C**)**. Analyses with the LC–MS/MS system were performed on the resultant supernatant.

### UHPLC-MS/MS analysis

Novogene Co., Ltd. (Beijing, China) performed the UHPLC-MS/MS analyses with a Vanquish UHPLC system and an Orbitrap Q Exactive TM HF mass spectrometer (Germany, Thermo Fisher). A 17-min linear gradient was used with a flow rate of 0.2 mL/min to inject samples onto a 100 × 2.1 mm Hypesil Goldcolumn (1.9 μm). Eluent A (0.1% formic acid in Water) and Eluent B (Methanol) were used in the positive polarity mode. Eluent A with 5 mM ammonium acetate (pH 9.0) and B with methanol was used in the negative polarity mode. It was decided that the following gradient of solvent concentrations would be used: 2% B, 1.5 min; 2% -100%B, 12.0 min; 100% B, 14.0 min; 100–2% B, 14.1 min; 2% B, 17 min. We used the Q Exactive TM HF mass spectrometer for this experiment, set it to spray voltage mode, and ran at a capillary temperature of 320 °C with a sheath gas flow rate of 40 arb. We also used an aux gas flow rate of 10 arb.

### Metabolome analysis

To evaluate and validate the differences and reliability of metabolites in the samples, principal component analysis (PCA) was used. To search for differential metabolites, significant difference criteria [variable importance in projection (VIP) ≥ 1 and t-test p < 0.05] were employed. The Kyoto Encyclopedia of Genes and Genomes (KEGG) database was used to investigate the function of the differential metabolites and metabolic pathways. Differentially accumulated metabolites (DAMs) were screened based on fold-change (FC) ≥ 2 or ≤ 0.5 among the metabolites with a VIP value > 1 and a P-value < 0.05. The top 20 differentially (up and down) produced metabolites in BFC and BFT were analyzed to find the major differential metabolites and steroidal alkaloids. Heat maps were used to figure out how the main steroid alkaloids in BFC and BFT were different.

### RNA Sequencing

Six species' bulbs were used to extract the total mRNA. Each sample's mRNA library was constructed. The index-coded samples were clustered using the TruSeq PE Cluster Kit v3-cBot-HS (Illumia) on a cBot Cluster Generation System. Each sample had six biological replicates. Raw fastq data (raw reads) was first processed by in-house Perl scripts. This step removed adapter, ploy-N, and low-quality reads from raw data. Simultaneously, Q20, Q30, and GC content were calculated. All downstream analyses used clean, high-quality data.

### Functional annotation and screening of differentially expressed genes (DEGs)

These databases used to annotate the gene function included the Nr (NCBI non-redundant protein sequences), Nt (NCBI non-redundant nucleotide sequences), Pfam (Protein Family), KO (KEGG Ortholog database), and GO (Gene Ontology). The DESeq2R package was used to compare differential expression between two conditions/groups (two biological replicates each) (1.20.0). DESeq2 classified genes as differentially expressed if their adjusted P-value was less than 0.05. In the cluster Profiler R package, gene length bias was corrected before performing GO enrichment analysis on differentially expressed genes. Derived from the GO database, differentially expressed genes were considered to be significant. We used the R package cluster Profiler to perform KEGG pathway enrichment of differentially expressed genes. We found the GO terms or KEGG pathways involved alkaloids biosynthesis that was significantly enriched in DEGs compared to BFC and BFT. The biosynthetic route for BFC steroidal alkaloids was hypothesized based on an analysis of the chemical structures of several kinds of steroid alkaloids and the role of their biocatalytic enzymes in functionalizing the steroid skeleton. Structural genes associated with alkaloid synthesis were identified by comparing significantly different genes. TFs genes upregulated in two or more of the five BFC species were selected to regulate the alkaloid synthesis.

### Quantitative Reverse Transcription Polymerase Chain Reaction (qRT-PCR) for Verification of the RNA-Seq Data

qRT-PCR was used to validate the expression levels of the candidate genes in the proposed biosynthetic pathway of steroidal alkaloids. The primer sequences were shown in Supplemental Table S5. Ct values were calculated from 3 biological replicates and 3 technical replicates. The Ct value determined the expression level of the reference genes, and the expression level of the reference gene was calculated by 2 − △△Ct.

## Supplementary Information

**Additional file 1.**

## Data Availability

The raw RNA-seq read data are deposited in the Short Read Archive (http://www.ncbi.nlm.nih.gov/sra/) and are accessible in GenBank through accession number (BioProject PRJNA765152, PRJNA765165, PRJNA765176, PRJNA765126, PRJNA765399, PRJNA765438). Metabolome data and all unigenes obtained in our trancriptome sequencing are available on FigShare at the link: https://doi.org/10.6084/m9.figshare.19932038.v1.

## Abbreviations

IPP: Isopentenyl diphosphate
DMAPP: Dimethylallyl diphosphate
MVA: Mevalonic acid
MEP: Methylerythritol 4-phosphate
RT-qPCR: Reverse transcription-quantitative polymerase chain reaction
JY: *Fritillaria cirrhosa*
GS: *Fritillaria przewalskii*
SS: *Fritillaria delavayi*
TB: *Fritillaria taipaiensis*
WB: *Fritillaria unibracteata*
ZB: *Fritillaria thunbergii*
LC − MS/MS: Liquid chromatography-tandem mass spectrometry
PCA: Principal component analysis
VIP: Variable importance in projection
KEGG: The Kyoto Encyclopedia of Genes and Genomes
DEGs: Differentially expressed genes
Nr: NCBI non-redundant protein sequences
Nt: NCBI non-redundant nucleotide sequences
Pfam: Protein Family
KO: KEGG Ortholog database
GO: Gene Ontology
qRT-PCR: Quantitative Reverse Transcription Polymerase Chain Reaction
DAMs: Differentially accumulated metabolites
FC: Fold-change
BP: Biosynthetic process
GPP: Geranyl pyrophosphate
FPP: Farnesyl pyrophosphate
FPPS: Farnesyl pyrophosphate synthase
SQS: Squalene synthase
SQE: Squalene monooxygenase
CAS: Cycloartenol synthase
SSR2: Sterol side chain reductase
3βHSD: 3-Beta-hydroxysteroid-dehydrogenase/decarboxylase
CPI: Cyclopropyl isomerase
CYP51: Sterol 14-demethylase
C5-SD: Sterol C-5 desaturase
7-DR: 7-Dehydrocholesterol reductase
TFs: Transcription factors
SGA: Steroidal glycoalkaloids
