# Supplementary material for: Integrative analysis of the steroidal alkaloids distribution and biosynthesis of bulbs Fritillariae Cirrhosae through metabolome and transcriptome analyses
Source: BMC Genomics. 2022 Jul 14;23:511. doi: 10.1186/s12864-022-08724-0 (PMC9284883; doi:10.1186/s12864-022-08724-0)
Supplement: Supplementary file 1 — Additional file 1. [file 12864_2022_8724_MOESM1_ESM.zip › Supplemental Files/Supplemental figures and tables.docx]

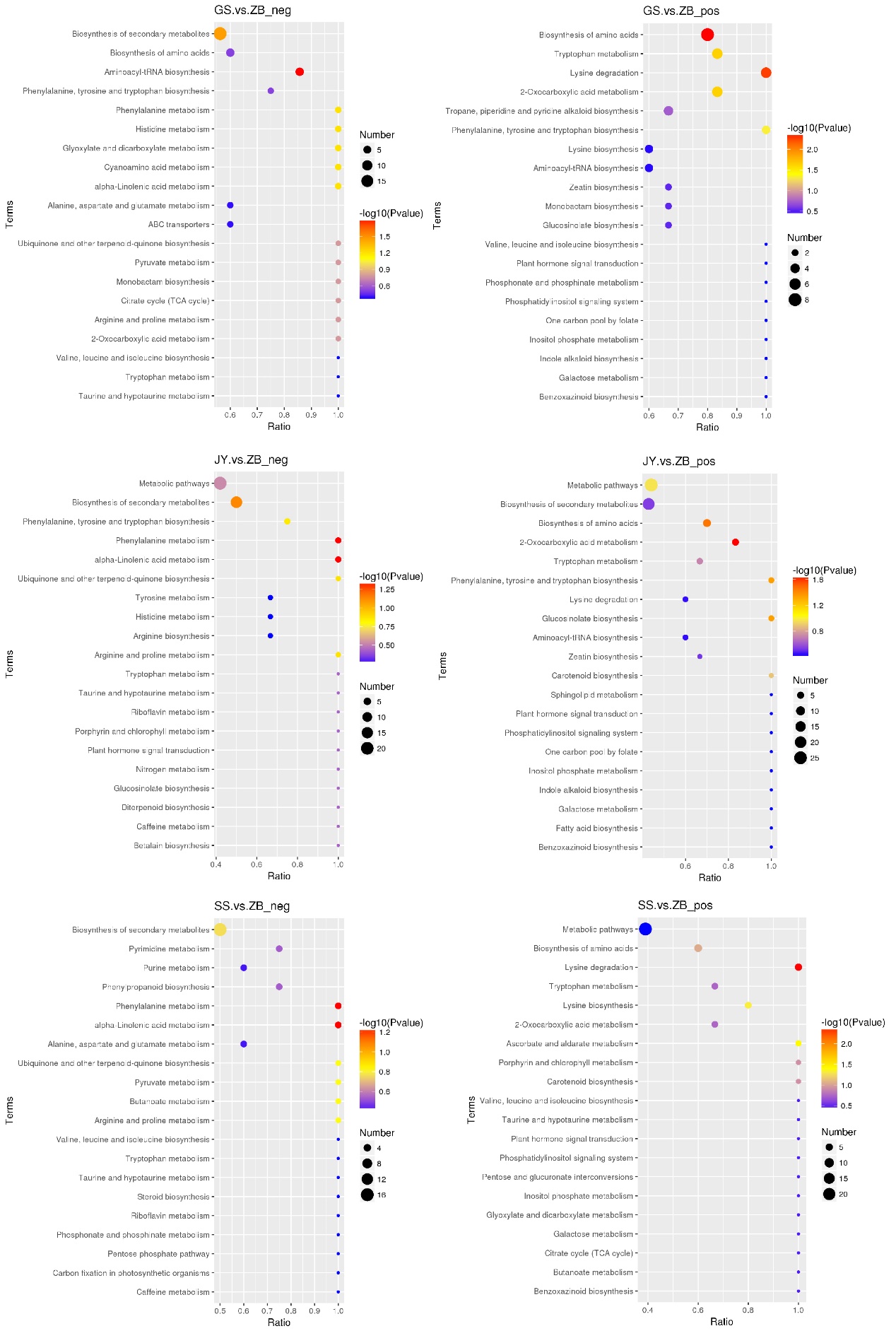


­­­­

**
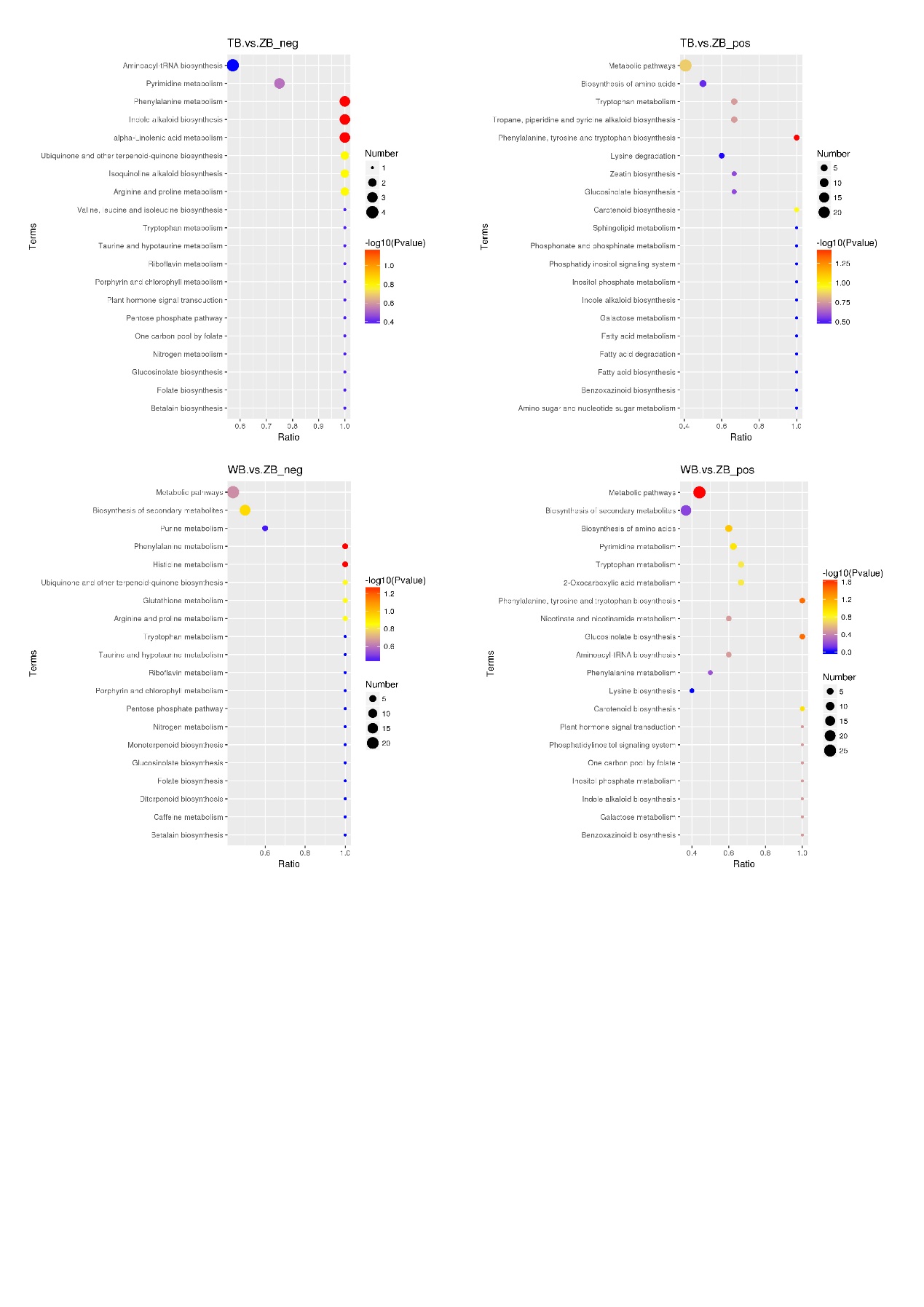
**

**Fig. S1.** KEGG enrichment results of differential metabolites in *Fritillaria cirrhosa* and *Fritillaria thunbergii.* (*Fritillaria przewalskii* (GS), *Fritillaria cirrhosa* D.Don (JY), *Fritillaria delavayi* (SS), *Fritillaria taipaiensis* (TB), *Fritillaria unibracteata* (WB) and *Fritillaria thunbergii* (ZB))

**
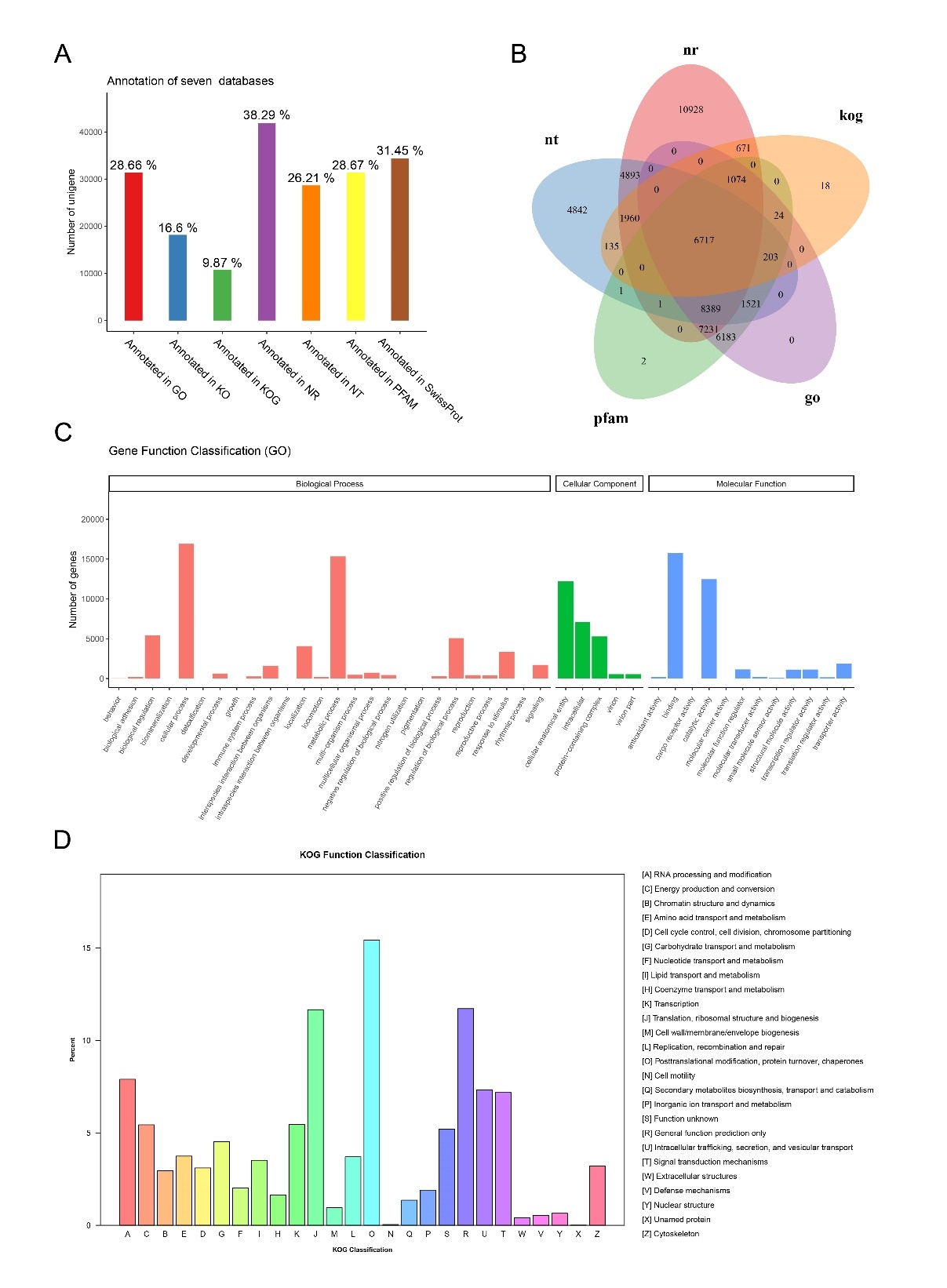
**

**Fig. S2.** Summary of annotations on unigenes against public databases. A. annotation of unigenes based on different databases; **B**. comparison of numbers of annotated unigenes against different databases; **C**. Gene ontology database (GO) classification of unigenes in five species of *Fritillaria cirrhosa* compared to *Fritillaria thunbergii;* **D**. KEGG pathway enrichment analysis of *Fritillaria cirrhosa* compared to *Fritillaria thunbergii*.


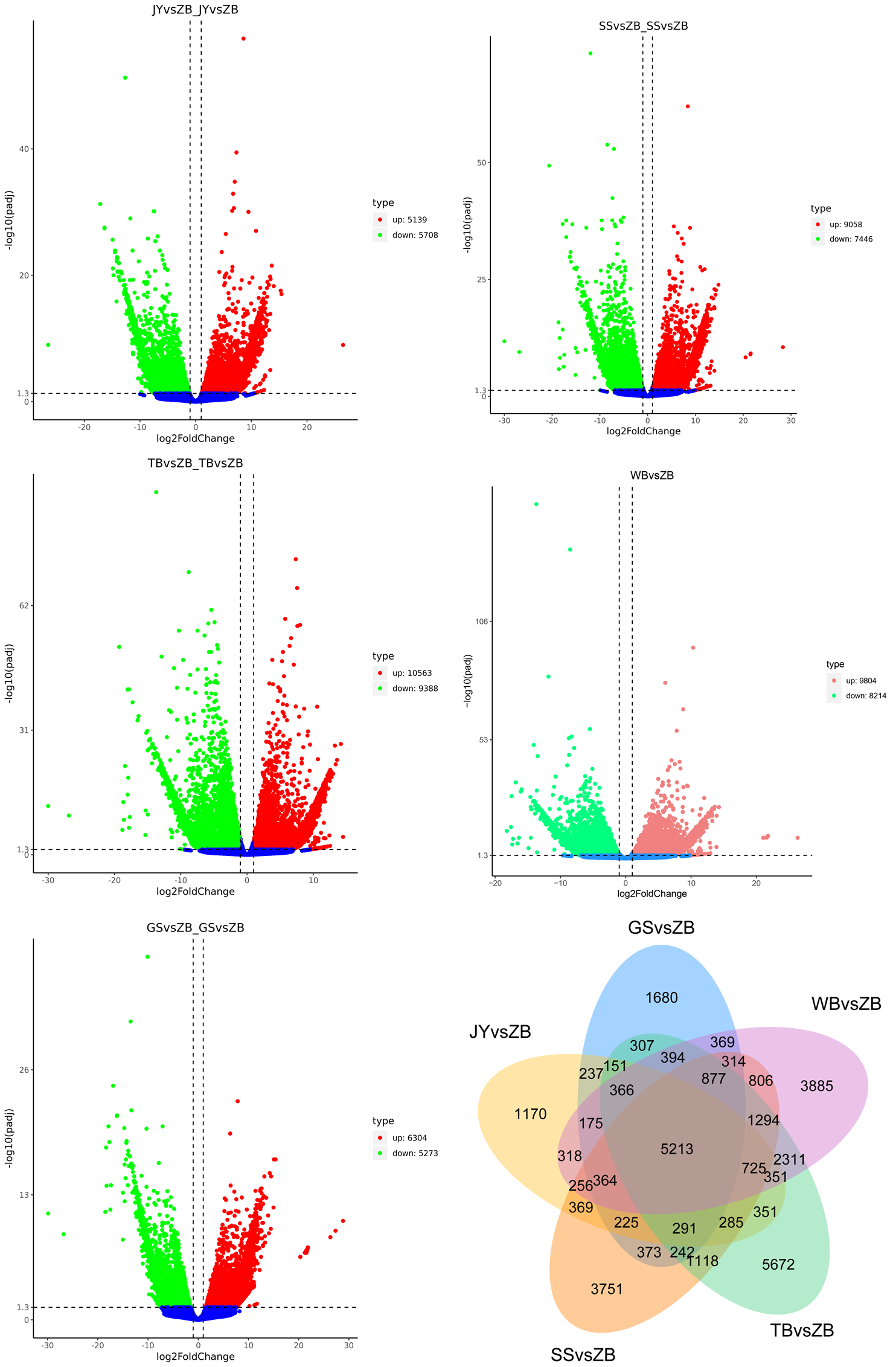


E

F

C

D

B

A

**Fig. S3.** Identification of the differentially genes between bulbs of *Fritillaria cirrhosa* and *Fritillaria thunbergii*. **A-E**. Volcano map between group comparisons; **F**. Venn diagram depicting the shared and unique differentially genes between group comparisons. (*Fritillaria cirrhosa* (JY), *Fritillaria delavayi* (SS), *Fritillaria taipaiensis* (TB), *Fritillaria unibracteata* (WB), *Fritillaria przewalskii* (GS), and *Fritillaria thunbergii* (ZB))

A


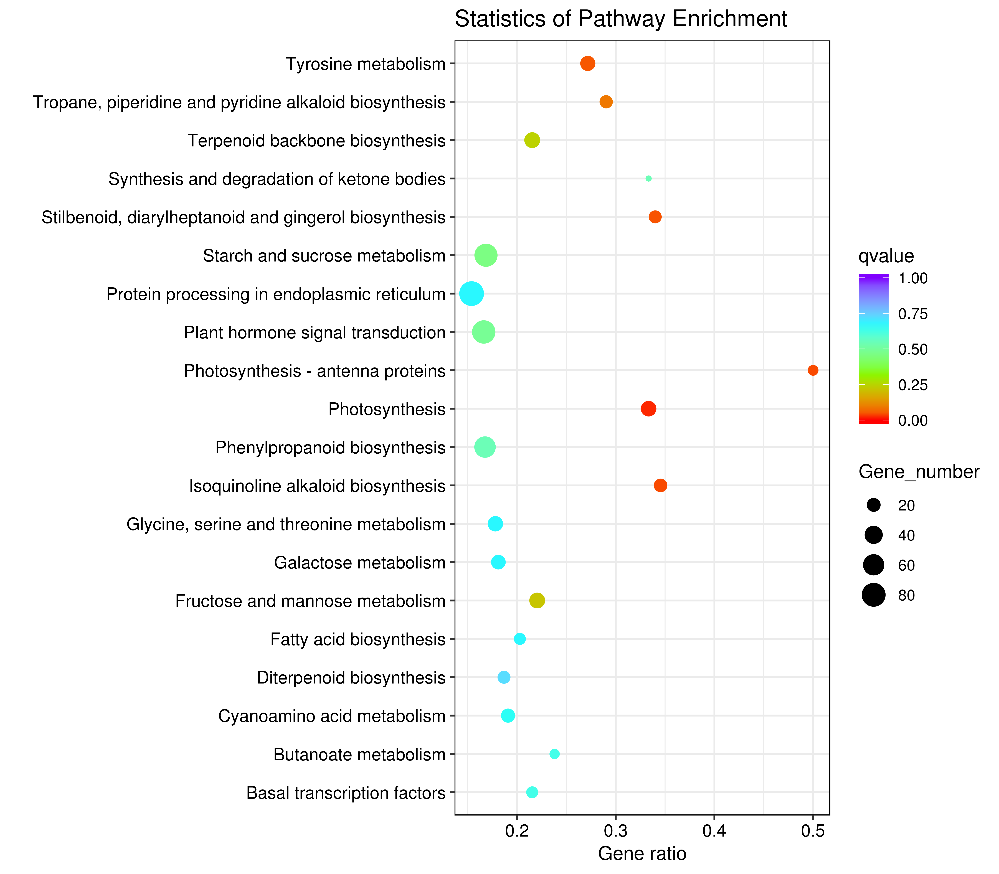


B


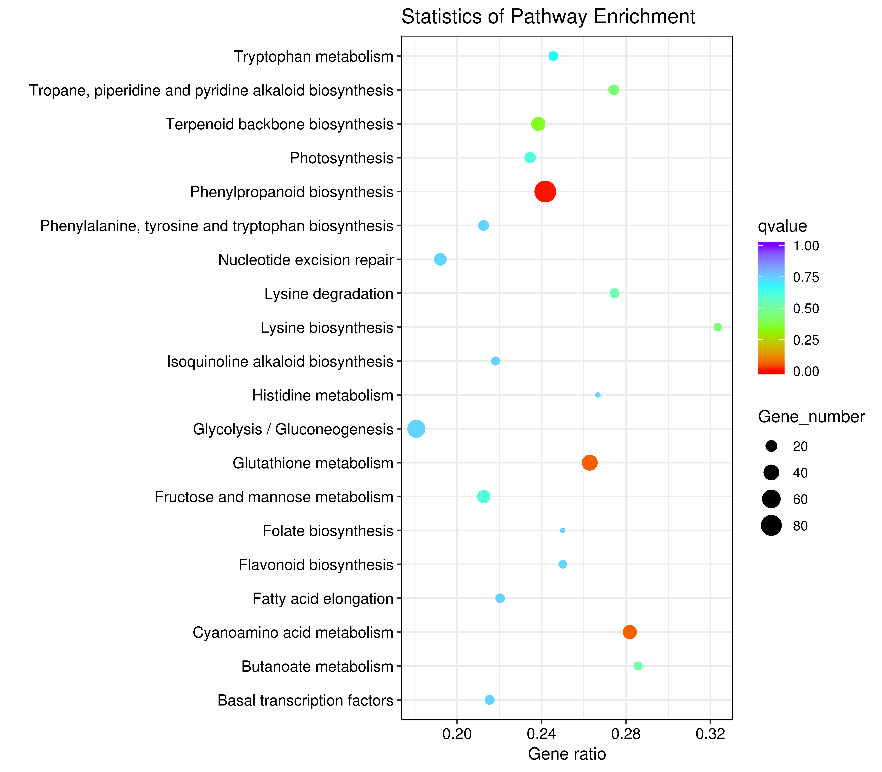


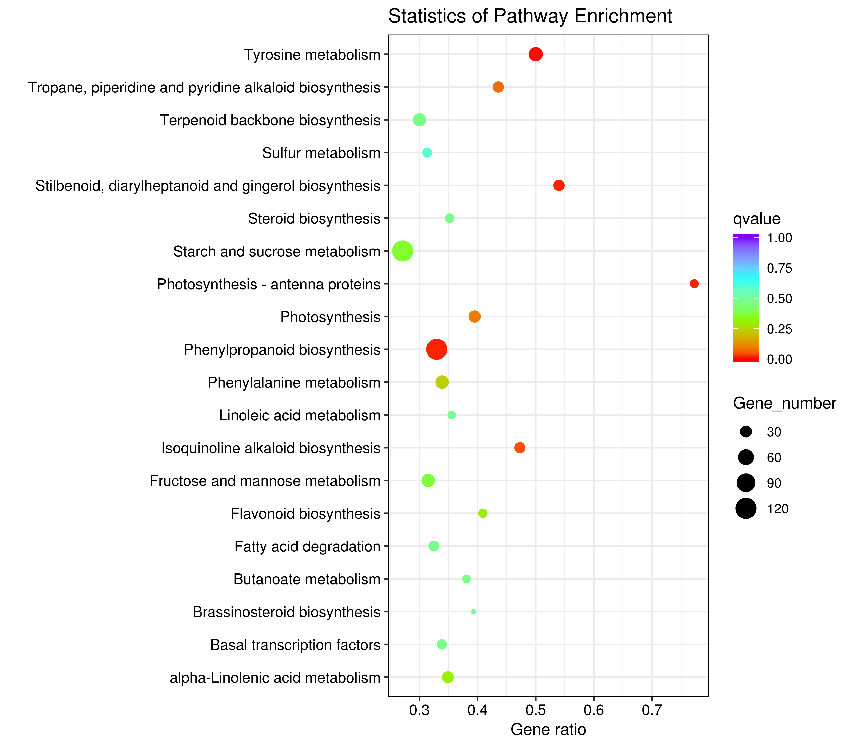


C

D


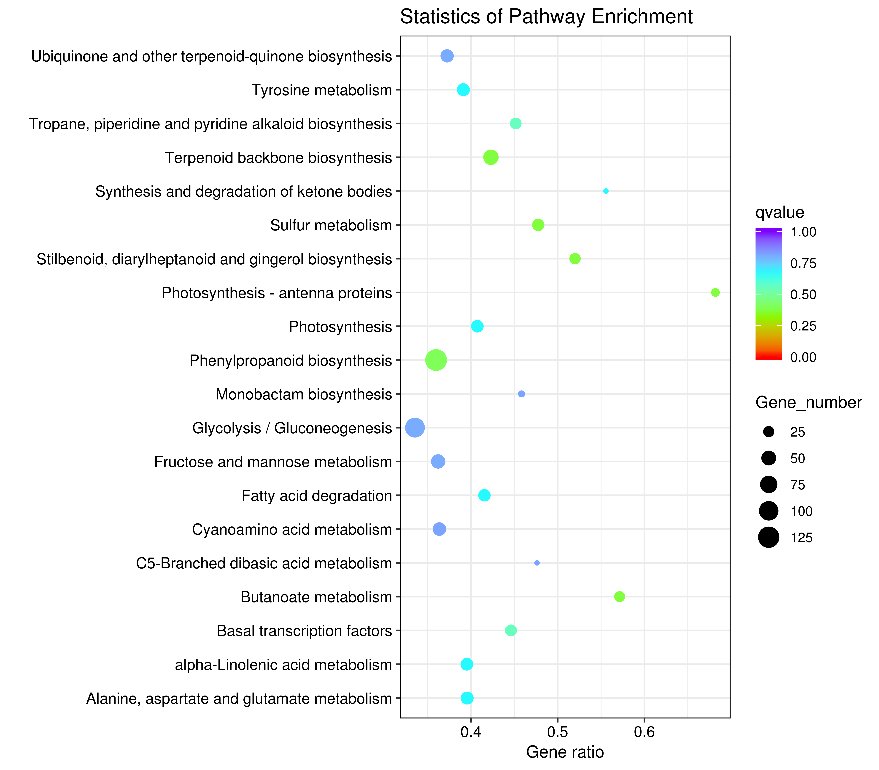


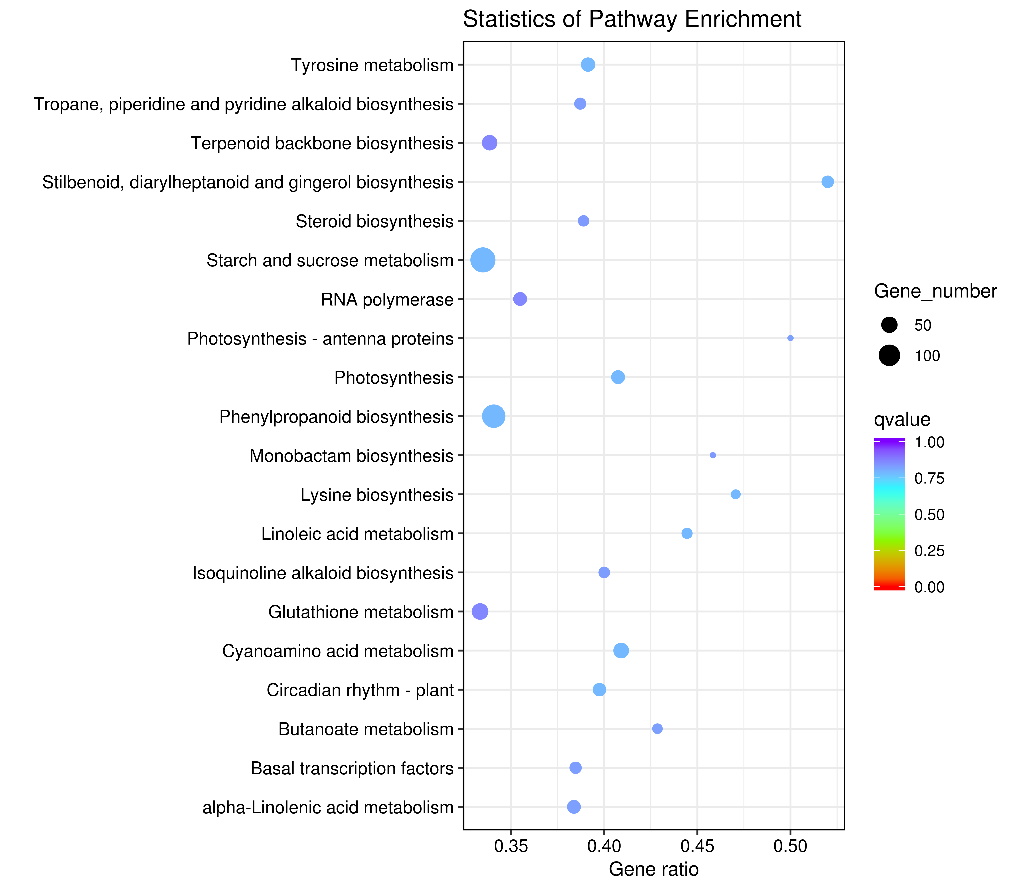


E

**Fig. S4**. Top 20 of differential genes for KEGG enrichment in *F. cirrhosa* and *F. thunbergii*. **A.** *Fritillaria cirrhosa* (JY) vs *Fritillaria thunbergii* (ZB); **B.** *Fritillaria delavayi* (SS) vs *Fritillaria thunbergii* (ZB); **C.** *Fritillaria taipaiensis* (TB) vs *Fritillaria thunbergii* (ZB); **D.** *Fritillaria unibracteata* (WB) vs Fritillaria thunbergii (ZB); **E.** *Fritillaria przewalskii* (GS) vs *Fritillaria thunbergii* (ZB). The ordinate is pathway. The horizontal axis is the enrichment factor (the number of differences in this Pathway divided by all Numbers). The size is the quantity, the redder the color, the smaller the P/Q value.


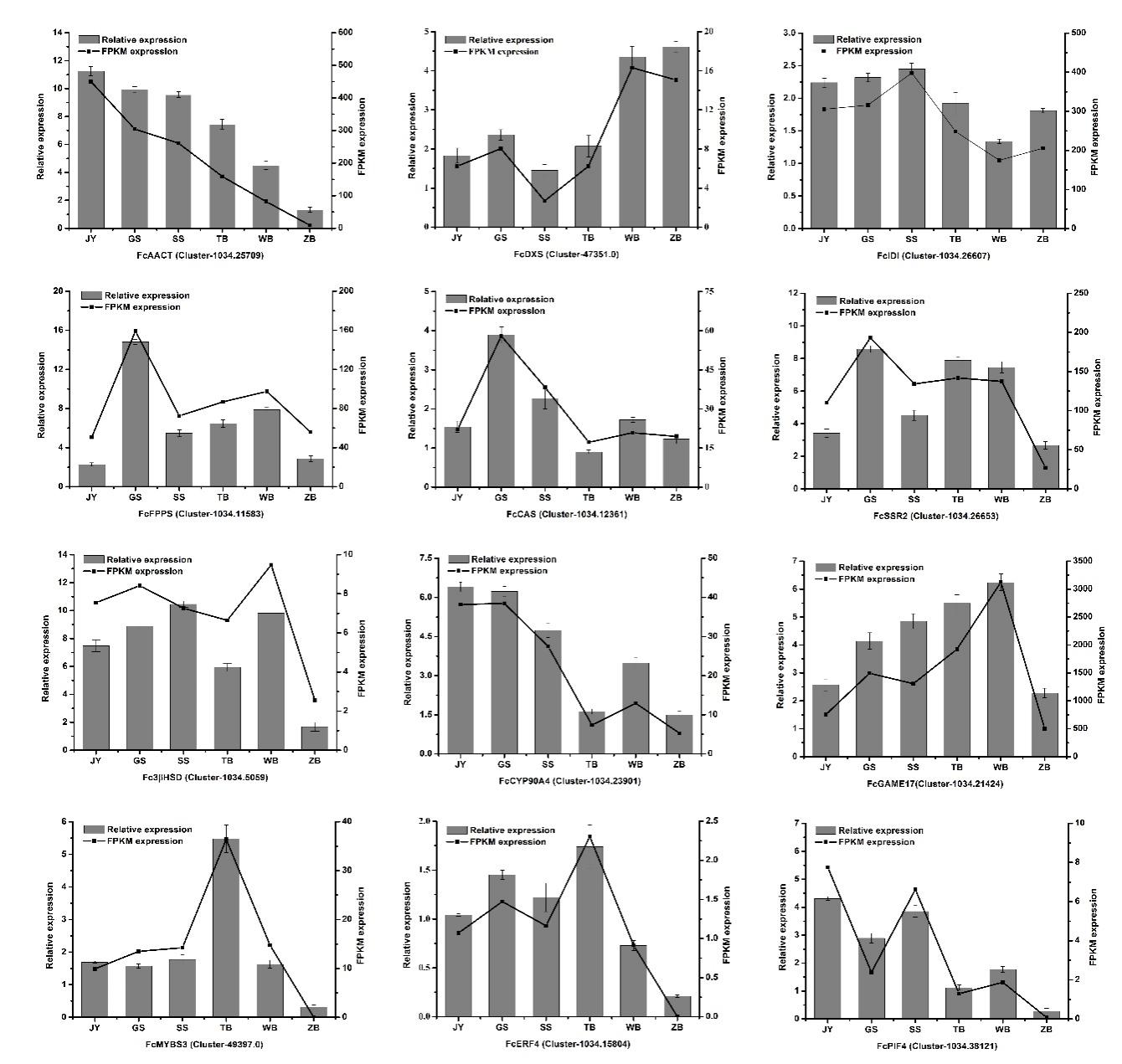


**Fig. S5.** Expression analysis of structural genes and transcription factors related to the steroidal alkaloid biosynthesis pathway: analysis of expression levels of key structural genes and transcription factors during steroid alkaloids biosynthesis in *Fritillaria cirrhosa* and *Fritillaria thunbergii.*

Table S1. The Top 20 Differentially (up and down) produced metabolites

| Compounds | Class | VIP | Fold_Change | Log2FC | type |
| --- | --- | --- | --- | --- | --- |
| **JY vs ZB** |  |  |  |  |  |
| Ginkgolide C | Terpenoids | 1.51 | 5820.05 | 12.51 | up |
| Khasianine | Alkaloids | 2.00 | 1378.10 | 10.43 | up |
| 4-Coumaric acid | Prenol lipids | 1.40 | 340.41 | 8.41 | up |
| Trigoneoside Xb | Alkaloids | 1.06 | 267.12 | 8.06 | up |
| gamma-Glutamyltyrosine | Carboxylic acids and derivatives | 1.92 | 230.89 | 7.85 | up |
| Flunitrazepam-d3 | Benzodiazepine | 1.95 | 226.98 | 7.83 | up |
| Edpetiline | Alkaloids | 1.83 | 198.86 | 7.64 | up |
| Bilobalide | Prenol lipids | 1.25 | 180.98 | 7.50 | up |
| Quinic acid | Organic oxygen compounds | 1.33 | 152.96 | 7.26 | up |
| L-2-Aminoadipic acid | Carboxylic acids and derivatives | 2.17 | 114.52 | 6.84 | up |
| Calceolarioside B | Phenylpropanoids | 1.38 | 114.28 | 6.84 | up |
| Glibenclamide | Alkaloids | 1.27 | 97.26 | 6.60 | up |
| Polygalaxanthon VIII | others | 1.15 | 96.25 | 6.59 | up |
| Sibirioside A | Phenylpropanoids | 1.50 | 95.85 | 6.58 | up |
| Emamectin B1a | others | 1.45 | 77.92 | 6.28 | up |
| Homoharringtonine | Alkaloids | 1.75 | 88.09 | 6.46 | up |
| beta-Nicotyrine | Alkaloids | 1.30 | 73.75 | 6.20 | up |
| Tryptamine | Alkaloids | 1.23 | 61.20 | 5.94 | up |
| Pilocarpine | Alkaloids | 1.84 | 50.44 | 5.65 | up |
| Tomatidenol | Alkaloids | 1.90 | 46.23 | 5.53 | up |
| 4-(4-cyclohexylphenyl)-4-oxobut-2-enoic acid | others | 2.11 | 0.001 | -8.98 | down |
| 3',6-Disinapoyl sucrose | Glycolipids | 2.06 | 0.004 | -7.92 | down |
| L-Saccharopine | Carboxylic acids and derivatives | 2.07 | 0.004 | -7.89 | down |
| Peimine | Alkaloids | 1.76 | 0.005 | -7.42 | down |
| 1,2-dihydroxyheptadec-16-yn-4-yl acetate | Fatty alcohols | 2.11 | 0.006 | -7.29 | down |
| Sinapoyl O-hexoside | others | 1.98 | 0.009 | -6.69 | down |
| Gibberellin A7 | Prenol Lipids | 1.59 | 0.010 | -6.60 | down |
| Lapachone | Quinones | 1.82 | 0.020 | -5.65 | down |
| 13(S)-HOTrE | Lipids_Fatty acids | 1.52 | 0.023 | -5.43 | down |
| 10-Deacetylbaccatin-III | others | 2.05 | 0.025 | -5.33 | down |
| Deoxyadenosine | Nucleosides, nucleotides, and analogues | 1.79 | 0.035 | -4.99 | down |
| 4-Dimethylaminobenzaldehyde | others | 2.22 | 0.032 | -4.96 | down |
| Nardosinone | Terpenoids | 2.16 | 0.032 | -4.94 | down |
| Thymidine | Nucleosides, nucleotides, and Analogues | 1.75 | 0.032 | -4.95 | down |
| Sildenafil-d3 | Vitamin | 2.06 | 0.033 | -4.93 | down |
| Cordycepin | Alkaloids | 1.88 | 0.035 | -4.82 | down |
| Santacruzamate A | others | 2.04 | 0.036 | -4.79 | down |
| Lysops 22:6 | Lipids | 1.98 | 0.052 | -4.26 | down |
| 12-Oxo phytodienoic acid | Organic acids and derivatives | 1.75 | 0.065 | -3.95 | down |
| Lawsone | Benzenoids | 1.73 | 0.065 | -3.94 | down |
| **SS vs ZB** |  |  |  |  |  |
| Ginkgolide C | Anthocyanins | 1.88 | 3763.05 | 11.88 | up |
| Solamargine | Alkaloids | 1.90 | 2455.02 | 11.26 | up |
| Khasianine | Alkaloids | 1.95 | 1296.80 | 10.34 | up |
| Trigoneoside Xb | Alkaloids | 1.86 | 1150.86 | 10.17 | up |
| Solanine | Alkaloids | 1.99 | 955.92 | 9.90 | up |
| Protogracillin | Lipids | 1.73 | 714.74 | 9.48 | up |
| Solanidine | Alkaloids | 1.90 | 603.19 | 9.24 | up |
| Farnesyl pyrophosphate | Terpenoids | 1.59 | 435.33 | 8.77 | up |
| Edpetiline | Alkaloids | 1.96 | 286.60 | 8.16 | up |
| 4-Coumaric acid | Phenylpropanoids and polyketides | 1.77 | 236.25 | 7.88 | up |
| Flavokawain A | Chalcones | 1.85 | 176.86 | 7.47 | up |
| Astragaloside IV | Triterpenoids | 1.88 | 138.90 | 7.12 | up |
| Polygalaxanthon VIII | others | 1.76 | 125.43 | 6.97 | up |
| Calceolarioside B | Phenylpropanoids | 1.64 | 106.79 | 6.74 | up |
| Timosaponin A-III | Steroids | 1.84 | 104.05 | 6.70 | up |
| L-2-Aminoadipic acid | Organic acids and derivatives | 2.02 | 96.89 | 6.60 | up |
| Limocitrin O-hexoside | Flavonoids | 1.74 | 93.08 | 6.54 | up |
| Glibenclamide | Alkaloids | 1.40 | 77.08 | 6.27 | up |
| Polyphyllin VI | Steroids | 1.76 | 73.41 | 6.20 | up |
| Oridonin | Diterpenoids | 1.59 | 67.93 | 6.09 | up |
| Peimine | Alkaloids | 1.97 | 0.002 | -9.28 | down |
| L-Saccharopine | Organic acids and derivatives | 2.21 | 0.002 | -9.06 | down |
| 1,2-dihydroxyheptadec-16-yn-4-yl acetate | Fatty alcohols | 2.06 | 0.004 | -7.83 | down |
| Sinapoyl O-hexoside | Others | 2.13 | 0.006 | -7.36 | down |
| 3',6-Disinapoyl sucrose | Glycolipids | 1.57 | 0.015 | -6.06 | down |
| 10-Deacetylbaccatin-III | Terpenoids | 2.08 | 0.019 | -5.71 | down |
| 4-Dimethylaminobenzaldehyde | Others | 2.02 | 0.038 | -4.71 | down |
| Lapachone | Quinones | 1.42 | 0.041 | -4.60 | down |
| 2'-Deoxyadenosine | Nucleosides, nucleotides, and analogues | 1.55 | 0.045 | -4.47 | down |
| Sildenafil-d3 | Vitamin | 1.79 | 0.046 | -4.44 | down |
| Nardosinone | Terpenoids | 1.89 | 0.048 | -4.39 | down |
| Phellodensin F | Others | 1.91 | 0.049 | -4.36 | down |
| Thymidine | Nucleosides, nucleotides, and analogues | 1.49 | 0.050 | -4.32 | down |
| Cordycepin | Alkaloids | 1.59 | 0.055 | -4.19 | down |
| Phlorizin | Phenylpropanoids and polyketides | 1.99 | 0.059 | -4.07 | down |
| Prim-O-glucosylcimifugin | Terpenoids | 2.02 | 0.063 | -4.00 | down |
| Methyl linoleate | Lipids | 1.42 | 0.063 | -3.98 | down |
| Santacruzamate A | Others | 1.60 | 0.064 | -3.97 | down |
| 13(S)-HOTrE | Lipids | 1.19 | 0.065 | -3.94 | down |
| Lawsone | Benzenoids | 1.51 | 0.065 | -3.94 | down |
| **TB vs ZB** |  |  |  |  |  |
| Ginkgolide C | Terpenoids | 1.16 | 4447.91 | 12.12 | up |
| Platycodin D | Terpenoids | 1.72 | 1788.06 | 10.80 | up |
| Khasianine | Alkaloids | 1.42 | 334.96 | 8.39 | up |
| 4-Coumaric acid | Phenylpropanoids and polyketides | 1.15 | 300.36 | 8.23 | up |
| Citrulline | Alkaloids | 2.01 | 292.30 | 8.19 | up |
| Calceolarioside B | Phenylpropanoids and polyketides | 1.06 | 179.29 | 7.49 | up |
| Edpetiline | Alkaloids | 1.63 | 148.88 | 7.22 | up |
| Oleuropein | Terpenoids_ | 2.01 | 137.04 | 7.10 | up |
| Flunitrazepam-d3 | Benzodiazepine | 1.74 | 93.01 | 6.54 | up |
| PMeOH (18:2-18:3) | Lipids | 1.42 | 91.91 | 6.52 | up |
| Baohuoside II | Flavonoids | 1.92 | 60.72 | 5.92 | up |
| Eleutheroside E | Lignans | 2.00 | 52.71 | 5.72 | up |
| beta-Nicotyrine | Alkaloids | 1.39 | 36.20 | 5.18 | up |
| Polygalaxanthone IV | Others | 1.80 | 35.71 | 5.16 | up |
| Albiflorin | Terpenoids | 1.94 | 31.97 | 5.00 | up |
| Flavokawain B | Chalcones | 1.10 | 31.38 | 4.97 | up |
| Gamma-Glutamyltyrosine | Amino acids, peptides, and analogues | 1.40 | 30.42 | 4.93 | up |
| Quinic acid | Organic oxygen compounds | 1.14 | 30.26 | 4.92 | up |
| Sesamoside | Iridoids | 1.85 | 30.25 | 4.92 | up |
| Vindoline | Alkaloids | 1.50 | 28.99 | 4.86 | up |
| Peimine | Alkaloids | 2.02 | 0.003 | -8.15 | down |
| L-Saccharopine | Organic acids and derivatives | 1.89 | 0.007 | -7.13 | down |
| 3',6-Disinapoyl sucrose | Glycolipids | 1.78 | 0.011 | -6.44 | down |
| Sinapoyl O-hexoside | Others | 1.89 | 0.012 | -6.34 | down |
| 13(S)-HOTrE | Lipids | 1.47 | 0.023 | -5.45 | down |
| Lapachone | Quinones | 1.69 | 0.026 | -5.29 | down |
| Santacruzamate A | Others | 2.03 | 0.028 | -5.17 | down |
| 10-Deacetylbaccatin-III | Others | 1.82 | 0.028 | -5.14 | down |
| Alpha-Asarone | Phenylpropanoids | 1.85 | 0.028 | -5.14 | down |
| Sildenafil-d3 | Vitamin | 2.13 | 0.030 | -5.06 | down |
| 12-Oxo phytodienoic acid | Organic acids and derivatives | 1.99 | 0.033 | -4.91 | down |
| Ingenol-3-angelate | Terpenoids | 2.39 | 0.033 | -4.91 | down |
| Oxysophocarpine | Alkaloids | 2.20 | 0.035 | -4.85 | down |
| Pterosin G | Others | 1.88 | 0.036 | -4.79 | down |
| Nardosinone | Terpenoids | 2.11 | 0.037 | -4.75 | down |
| Lysops 22:6 | Lipids | 2.00 | 0.037 | -4.74 | down |
| 4-Dimethylaminobenzaldehyde | Others | 2.15 | 0.038 | -4.72 | down |
| Thymidine | Nucleosides, nucleotides, and analogues | 1.58 | 0.039 | -4.69 | down |
| Thromoboxane B1 | Others | 1.94 | 0.040 | -4.63 | down |
| Bakuchiol | Phenols | 1.46 | 0.042 | -4.58 | down |
| **WB vs ZB** |  |  |  |  |  |
| 2-Aminoadipic acid | Organic acids and derivatives | 1.54 | 853.02 | 9.74 | up |
| Lysops 22:6 | Lipids | 2.33 | 798.67 | 9.64 | up |
| trans-10-Heptadecenoic Acid | Lipids | 1.92 | 377.03 | 8.56 | up |
| 7-Methylxanthine | Nucleosides, nucleotides, and analogues | 2.12 | 249.84 | 7.96 | up |
| D-Phenylalanine | Alkaloids | 1.81 | 174.11 | 7.44 | up |
| Gibberellin A7 | Prenol Lipids | 1.26 | 122.92 | 6.94 | up |
| 4,5-DCQA Isochlorogenic acid C | Organoheterocyclic compounds | 1.96 | 97.98 | 6.61 | up |
| Naringin Dihydrochalcone | Chalcones | 1.95 | 96.30 | 6.59 | up |
| 3-Amino-4-methylpentanoic acid | Amino acids, peptides, and analogues | 1.96 | 88.63 | 6.47 | up |
| Deacetyltaxol | Terpenoids | 1.23 | 70.73 | 6.14 | up |
| Methyl oleate | Others | 1.16 | 62.13 | 5.96 | up |
| Aminobutyric acid (GABA) | Prenol lipids | 1.56 | 60.81 | 5.93 | up |
| Pentadecanoic acid | Lipids | 2.23 | 57.06 | 5.83 | up |
| trans-Cinnamic acid | Cinnamic acids and derivatives | 1.18 | 56.78 | 5.83 | up |
| 2-oxopiperidine-3-carbohydrazide | Organic acids and derivatives | 1.80 | 53.74 | 5.75 | up |
| 3',6-Disinapoyl sucrose | Glycolipids | 1.06 | 52.13 | 5.70 | up |
| Ginkgolic Acid | Phenols | 2.10 | 51.70 | 5.69 | up |
| gamma,gamma-Dimethylallyl pyrophosphate | Organic oxygen compounds | 1.34 | 50.05 | 5.65 | up |
| Sedoheptulose Anhydride | Organic acids and derivatives | 1.18 | 49.69 | 5.63 | up |
| Alpha-Linolenic acid | Lipids | 1.82 | 49.66 | 5.63 | up |
| 2-Aminoadipic acid | Organic acids and derivatives | 2.01 | 0.004 | -8.14 | down |
| Lysops 22:6 | Lipids | 1.91 | 0.004 | -7.86 | down |
| trans-10-Heptadecenoic Acid | Lipids | 1.59 | 0.007 | -7.14 | down |
| 7-Methylxanthine | Nucleosides, nucleotides, and analogues | 2.01 | 0.007 | -7.11 | down |
| LPE 20:5 | Lipids | 1.52 | 0.010 | -6.67 | down |
| D-Phenylalanine | Alkaloids | 2.28 | 0.020 | -5.61 | down |
| (+)-Camphor | Lipids | 2.18 | 0.022 | -5.54 | down |
| Naringin Dihydrochalcone | Chalcone | 2.11 | 0.027 | -5.23 | down |
| 3-Amino-4-methylpentanoic acid | Amino acids, peptides, and analogues | 2.12 | 0.027 | -5.23 | down |
| Deacetyltaxol | Terpenoids | 2.13 | 0.034 | -4.88 | down |
| Methyl oleate | Others | 2.20 | 0.035 | -4.85 | down |
| Pentadecanoic acid | Lipids | 1.76 | 0.038 | -4.72 | down |
| trans-Cinnamic acid | Cinnamic acids and derivatives | 2.05 | 0.042 | -4.56 | down |
| 2-oxopiperidine-3-carbohydrazide | Others | 1.49 | 0.043 | -4.54 | down |
| 3',6-Disinapoyl sucrose | Nucleosides, nucleotides, and analogues | 1.40 | 0.047 | -4.40 | down |
| Ginkgolic Acid | Phenols | 1.40 | 0.050 | -4.33 | down |
| Sedoheptulose Anhydride | Others | 1.91 | 0.051 | -4.28 | down |
| 11(Z),14(Z)-Eicosadienoic Acid | Lipids | 2.27 | 0.056 | -4.17 | down |
| Oleuropein | Terpenoids | 2.00 | 0.060 | -4.07 | down |
| 10-Deacetylbaccatin-III | Others | 1.35 | 0.065 | -3.94 | down |
| **GS vs ZB** |  |  |  |  |  |
| Khasianine | Alkaloids | 1.75 | 1053.62 | 10.04 | up |
| Coniferin | Organooxygen compounds | 1.55 | 754.01 | 9.56 | up |
| Flunitrazepam-d3 | Benzodiazepine | 1.96 | 242.80 | 7.92 | up |
| Citrulline | Alkaloids | 1.71 | 204.66 | 7.68 | up |
| gamma-Glutamyltyrosine | Organic acids and derivatives | 1.68 | 198.02 | 7.63 | up |
| PMeOH (18:2-18:3) | Lipids | 1.05 | 137.80 | 7.11 | up |
| beta-Nicotyrine | Alkaloids | 1.97 | 135.93 | 7.09 | up |
| L-2-Aminoadipic acid | Organic acids and derivatives | 2.13 | 130.82 | 7.03 | up |
| Tryptamine | Alkaloids | 1.98 | 128.89 | 7.01 | up |
| Indole | Alkaloids | 2.03 | 104.83 | 6.71 | up |
| 3-O-p-coumaroyl quinic acid O-hexoside | Quinate and its derivatives | 1.94 | 104.62 | 6.71 | up |
| 6-Methylquinoline | Alkaloids | 2.03 | 102.92 | 6.69 | up |
| L-Ergothioneine | Organic acids and derivatives | 1.94 | 101.83 | 6.67 | up |
| DL-Tryptophan | Alkaloids | 2.04 | 96.33 | 6.59 | up |
| LPA 16:0 | Lipids | 1.85 | 91.70 | 6.52 | up |
| Acetylcholine | Alkaloids | 1.79 | 62.92 | 5.98 | up |
| L-Phenylalanine | Alkaloids | 1.82 | 48.58 | 5.60 | up |
| Menadione | Benzenoids | 1.49 | 45.40 | 5.50 | up |
| Oleuropein | Terpenoids_ | 1.61 | 47.79 | 5.58 | up |
| 4-Hydroxymephenytoin | Coumarins | 1.84 | 44.38 | 5.47 | up |
| Peimine | Alkaloids | 2.03 | 0.001 | -9.47 | down |
| L-Saccharopine | Organic acids and derivatives | 2.03 | 0.003 | -8.36 | down |
| Sinapoyl O-hexoside | Others | 2.27 | 0.004 | -7.89 | down |
| 3',6-Disinapoyl sucrose | Glycolipids | 1.96 | 0.005 | -7.78 | down |
| 13(S)-HOTrE | Lipids | 1.51 | 0.016 | -5.96 | down |
| 10-Deacetylbaccatin-III | Others | 2.11 | 0.018 | -5.81 | down |
| Gibberellin A7 | Prenol Lipids | 1.40 | 0.024 | -5.36 | down |
| Lawsone | Benzenoids | 1.95 | 0.034 | -4.88 | down |
| Nardosinone | Terpenoids | 2.03 | 0.035 | -4.85 | down |
| Sildenafil-d3 | Vitamin | 1.89 | 0.037 | -4.74 | down |
| 4-Dimethylaminobenzaldehyde | Others | 1.93 | 0.042 | -4.57 | down |
| Oxysophocarpine | Alkaloids | 1.89 | 0.046 | -4.43 | down |
| Pterosin G | Others | 1.78 | 0.054 | -4.21 | down |
| Bullatine A | Alkaloids | 2.14 | 0.057 | -4.14 | down |
| Lysops 22:6 | Lipids | 1.93 | 0.057 | -4.12 | down |
| 8-iso-15-keto Prostaglandin E2 | Lipids | 1.83 | 0.058 | -4.11 | down |
| 3-hydroxyquinuclidine-3-carbonitrile hydrochloride | Others | 1.85 | 0.061 | -4.03 | down |
| N-Oleoyl dopamine | Lipids | 1.83 | 0.070 | -3.83 | down |
| Pseudoginsenoside-RT5 | Terpenoids | 1.42 | 0.076 | -3.72 | down |
| Ingenol-3-angelate | Terpenoids | 1.68 | 0.081 | -3.62 | down |

JY: *Fritillaria cirrhosa*; SS: *Fritillaria delavayi*; TB: *Fritillaria taipaiensis*;

WB: *Fritillaria unibracteata*; GS: *Fritillaria przewalskii*; ZB: *Fritillaria thunbergii*

**Table S2**. The content of peiminine in *F. cirrhosa* and *F. thunbergii.*

| species | concentration (mg/g) |
| --- | --- |
| WB | 0.099 |
| TB | 0.089 |
| JY | 0.078 |
| SS | 0.072 |
| GS | 0.041 |
| ZB | 0.077 |

JY: *Fritillaria cirrhosa*; SS: *Fritillaria delavayi*; TB: *Fritillaria taipaiensis*;

WB: *Fritillaria unibracteata*; GS: *Fritillaria przewalskii*; ZB: *Fritillaria thunbergii*

**Table S3** Numbers and quality of RNA-seq reads produced in each sample.

| Sample | NO. of Raw Reads | NO. of Clean Reads | Clean Bases | Q20(%) | Q30(%) | GC Content (%) |
| --- | --- | --- | --- | --- | --- | --- |
| JY_S1 | 23.94 | 23.51 | 7.05G | 98.5 | 95.54 | 50.68 |
| JY_S2 | 23.94 | 23.50 | 7.05G | 98.5 | 95.47 | 48.64 |
| JY_S3 | 26.25 | 25.65 | 7.69G | 98.22 | 94.93 | 48.24 |
| GS_S1 | 23.78 | 23.42 | 7.03G | 98.18 | 94.89 | 50.81 |
| GS_S2 | 23.71 | 23.33 | 7G | 98.19 | 94.87 | 50.06 |
| GS_S3 | 24.04 | 23.56 | 7.07G | 98.21 | 94.93 | 48.22 |
| SS_S1 | 25.65 | 25.24 | 7.57G | 98.18 | 94.87 | 50.41 |
| SS_S2 | 23.39 | 22.67 | 6.8G | 98.26 | 95 | 48.62 |
| SS_S3 | 21.57 | 21.15 | 6.35G | 98.32 | 95.15 | 49.54 |
| TB_S1 | 23.29 | 22.98 | 6.9G | 97.93 | 94.39 | 51.24 |
| TB_S2 | 22.90 | 22.62 | 6.79G | 98.07 | 94.68 | 50.7 |
| TB_S3 | 22.73 | 22.34 | 6.7G | 98.2 | 94.97 | 50.14 |
| WB_S1 | 21.57 | 21.34 | 6.4G | 98.12 | 94.75 | 51.06 |
| WB_S2 | 20.64 | 20.34 | 6.1G | 98.36 | 95.23 | 51.69 |
| WB_S3 | 22.67 | 22.31 | 6.69G | 98.42 | 95.34 | 51.01 |
| ZB_S1 | 23.79 | 23.30 | 6.99G | 98.26 | 95 | 47.52 |
| ZB_S2 | 22.55 | 22.17 | 6.65G | 98.23 | 94.9 | 47.9 |
| ZB_S3 | 25.28 | 24.86 | 7.46G | 98.22 | 94.91 | 48.37 |

Sample 1, Sample 2, and Sample 3 indicated three biological duplications.

**Table S4**Transcripts and fold changes of genes involved in the steroid alkaloid pathway

| Number | Name | Anotation | ID | Log2Fold change | | |  |  |
| --- | --- | --- | --- | --- | --- | --- | --- | --- |
|  |  |  |  | TB/ZB | WB/ZB | SS/ZB | GS/ZB | JY/ZB |
| 1 | *FcAACT* | Acetyl-CoA acetyltransferase | Cluster-1034.25709 | 3.9795 | 3.4441 | 5.0981 | 5.0691 | 5.5806 |
| 2 | *FcHMGS* | Hydroxymethylglutaryl-CoA synthase | Cluster-1034.19505 | 2.7484 | 2.6848 | 3.0808 | 3.808 | 2.9396 |
| 3 | *FcHMGR* | 3-hydroxy-3-methyl glutaryl coenzyme A reductase | Cluster-1034.25580 | 1.6354 | 2.6657 | 1.3863 | 1.9383 | 1.1765 |
| 4 | *FcMK* | Mevalonate kinase | Cluster-1034.9280 | 0.80588 | 1.1488 | 1.7142 | 1.1479 | 1.6008 |
| 5 | *FcPMK* | Phosphomevalonate kinase | Cluster-1034.31238 | -0.017561 | -0.46778 | 0.60509 | 0.21846 | -0.3446 |
| 6 | *FcMVD* | Diphosphomevalonate decarboxylase | Cluster-1034.34747 | -2.3995 | -1.7532 | 0.31823 | -0.01424 | -0.11814 |
| 7 | *FcDXS* | 1-deoxy-D-xylulose-5-phosphate synthase | Cluster-47351.0 | -1.4688 | 0.23318 | -2.3393 | -0.83482 | -1.3849 |
| 8 | *FcDXR* | chloroplast 1-deoxy-D-xylulose-5-phosphate reductoisomerase | Cluster-1034.20424 | -1.8076 | -2.7813 | -2.5724 | -3.1675 | -2.016 |
| 9 | *FcMCT* | 2-C-methyl-D-erythritol 4-phosphate cytidylyltransferase | Cluster-1034.20772 | -3.353 | -3.4191 | -2.6896 | -2.9319 | -2.2462 |
| 10 | *FcCMK* | 4-diphosphocytidyl-2-C-methyl-D-erythritol kinase | Cluster-1034.34336 | -2.1303 | -1.7092 | -0.64442 | -1.8496 | -0.86964 |
| 11 | *FcMCS* | 2-C-methyl-D-erythritol 2,4-cyclodiphosphate synthase | Cluster-1034.12325 | -2.4781 | -3.9998 | -2.6304 | -1.9517 | -1.3171 |
| 12 | *FcHDS* | 4-hydroxy-3-methylbut-2-en-1-yl diphosphate synthase | Cluster-1034.9543 | -2.7595 | -2.4001 | -3.0291 | -3.0628 | -3.0434 |
| 13 | *FcHDR* | 4-hydroxy-3-methylbut-2-enyl diphosphate reductase | Cluster-1034.31766 | 0.45214 | -0.52865 | -1.3245 | -0.54376 | -0.66364 |
| 14 | *FcIDI* | isopentenyl diphosphate isomerase | Cluster-1034.26607 | 0.063567 | -0.11773 | 1.0098 | 0.71091 | 0.44734 |
| 15 | *FcFPPS* | Farnesyl pyrophosphate synthase | Cluster-1034.11583 | 0.48425 | 1.1282 | 0.44944 | 1.4238 | -0.22225 |
| 16 | *FcSQS* | squalene synthase | Cluster-1034.11567 | -1.8401 | -1.4701 | -1.7157 | -1.1501 | -1.7986 |
| 17 | *FcSQE* | Squalene monooxygenase | Cluster-1034.20716 | 4.2935 | 3.7711 | 3.8491 | 3.8749 | 3.575 |
| 18 | *FcCAS* | cycloartenol synthase | Cluster-1034.12361 | -0.37659 | 0.37569 | 1.0172 | 1.4533 | 0.093458 |
| 19 | *FcSSR2* | sterol side chain reductase | Cluster-1034.26653 | 2.2309 | 2.6755 | 2.4334 | 2.8433 | 1.9535 |
| 20 | *Fc3βHSD* | 3beta-hydroxysteroid-dehydrogenase/decarboxylase | Cluster-1034.5059 | 1.2324 | 2.3161 | 1.5567 | 1.725 | 1.4934 |
| 21 | *FcCPI* | Cyclopropyl isomerase | Cluster-1034.28671 | 0.92701 | 0.82458 | 1.4906 | 1.3038 | 0.89554 |
| 22 | *FcCYP51* | sterol 14-demethylase | Cluster-1034.18398 | 0.65249 | 2.2609 | 1.6365 | 2.0805 | 1.3309 |
| 23 | *FcC5-SD* | sterol C-5 desaturase | Cluster-1034.10024 | 2.7719 | 2.2374 | 2.1254 | 2.3103 | 2.5772 |
| 24 | *Fc7-DR* | 7-dehydrocholesterol reductase | Cluster-1034.21535 | 3.3934 | 2.4522 | 3.4408 | 4.8026 | 4.6293 |
| 25 | *FcCYP90B1* | steroid 22-alpha-hydroxylase | Cluster-1034.31647 | -1.3483 | 4.7374 | 2.1394 | 5.918 | 6.1294 |
| 26 | *FcCYP734A6* | steroid 26-alpha-hydroxylase | Cluster-1034.31945 | -1.0613 | -0.49862 | 0.53058 | -0.96858 | 0.32629 |
| 27 | *FcCYP94N2* | steroid 22,26-alpha-hydroxylase | Cluster-1034.19824 | -1.382 | 1.6519 | -0.29994 | 1.2376 | 1.8842 |
| 28 | *FcCYP90A4* | Steroid 23-alpha-hydroxylase | Cluster-1034.23901 | 0.32056 | 1.6354 | 2.4553 | 2.8865 | 2.8088 |
| 29 | *FcAOP2* | 2-oxoglutarate-dependent  dioxygenase | Cluster-21667.1 | 0.64959 | 6.515 | 6.0864 | 4.0196 | 7.5495 |
| 30 | *FcGAME17* | UDP-glucose glucosyltransferase | Cluster-1034.21424 | 1.6805 | 2.7453 | 1.3781 | 1.6108 | 0.39249 |
| 31 | *FcGAME12* | Gamma-aminobutyrate  Aminotransferase | Cluster-50842.0 | 3.5436 | 5.0838 | 4.3547 | 3.8772 | 6.4203 |

Table S5. Primers for qRT-PCR analysis.

| Unigene ID | Gene annotation | Forward primer (5' to 3') | Reverse primer (5' to 3') | Product size (bp) |
| --- | --- | --- | --- | --- |
| Cluster-1034.25709 | AACT (acetyl-CoA acetyltransferase) | TGATGTTGTTGTGGCTGGTG | CACAAGCCATCTTTGAGCAT | 126 |
| Cluster-47351.0 | DXS (1-deoxy-D-xylulose-5-phosphate synthase) | GTTGTCACTGAAAAAGGGCG | AGCGGCGGAGGAAGTAGTTT | 238 |
| Cluster-1034.26607 | IDI (isopentenyl diphosphate isomerase) | TATCTCCTCTTCATCGTCCGT | GCCTTCCTCAGCAGTTCTTTC | 107 |
| Cluster-1034.11583 | FPPS (farnesyl pyrophosphate synthase) | ATTTCAAGTGTTCCTGGCTCA | ACCCTCATACTCCGCAAATAC | 167 |
| Cluster-1034.12361 | CAS (cycloartenol synthase) | CAGAGCGATGCCTTTAGTCCT | CTTTTTCAACCGCAGACCAC | 70 |
| Cluster-1034.26653 | SSR2 (sterol side chain reductase) | ACCTCCTTCTCGGTCTTC | TCCAATCTGGCTCTGTCC | 387 |
| Cluster-1034.5059 | 3Βhsd (3beta-hydroxysteroid-dehydrogenase/decarboxylase) | TTGATTGGTGTTGGGTTGATG | TTCTTTTGAATGTATGTCCCTATG | 167 |
| Cluster-1034.23901 | CYP90A4 (Steroid 23-alpha-hydroxylase) | TTGGTTTCGCTGCTTGTTG | AGGGCTCTGACTGGTCATTTT | 151 |
| Cluster-1034.21424 | GAME17 (UDP-glucose glucosyltransferase) | TCCACCTGGTTCTCCGTCTG | GGGGAATGCCCTCCTTATCTT | 141 |
| Cluster-49397.0 | MYBS3 (MYB transcription factor S3) | GGCTTGCCACTTGGGTAGGG | GGACCGAGGAGGAGCACAGAC | 117 |
| Cluster-1034.15804 | ERF4 (AP2/ERF transcription factor 4) | CTAACTGGAGGTGGCTGGGTAT | TCGGCATTAAACAAGGCAAA | 100 |
| Cluster-1034.3812137 | PIF4 (Phytochrome-interacting factor 4) | GCAACACTGCCTCCTAAATCTG | AACCACCCGAGGACGAAGTA | 340 |
| AY616727.1 | 18S ribosomal RNA gene | TACGACTCTCGGCAACGGA | CAAAGGGGCAATGGGAACA | 193 |
